# Correlative In Situ Spectro-Microscopy of Supported Single CuO Nanoparticles: Unveiling the Relationships between Morphology and Chemical State during Thermal Reduction

**DOI:** 10.1021/acsnano.4c01460

**Published:** 2024-05-14

**Authors:** Lucas de Souza Caldas, Mauricio J. Prieto, Liviu C. Tănase, Aarti Tiwari, Thomas Schmidt, Beatriz Roldan Cuenya

**Affiliations:** Department of Interface Science, Fritz-Haber Institute of the Max-Planck Society, Berlin 14195, Germany

**Keywords:** copper oxide, supported nanoparticles, PEEM, XAS, LEEM, thermal reduction, XPS

## Abstract

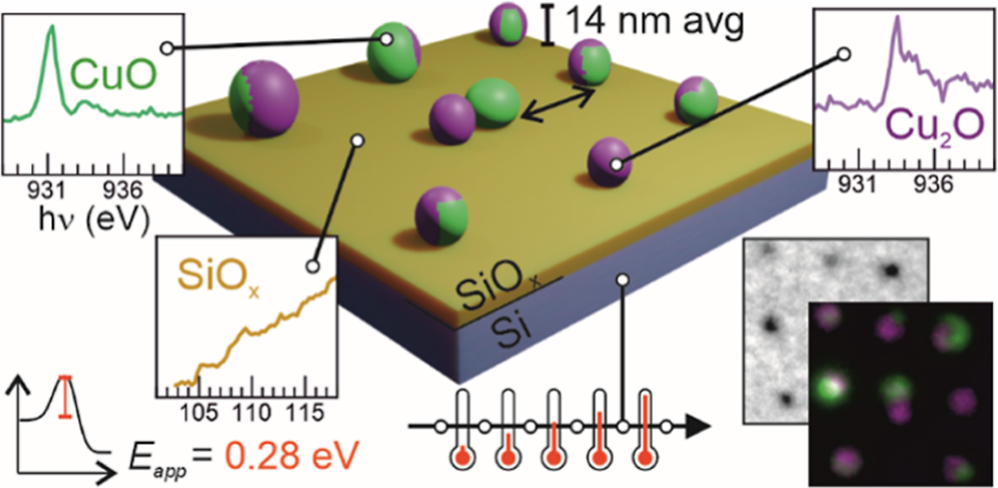

The activity, selectivity, and lifetime of nanocatalysts critically depend on parameters such as their morphology, support, chemical composition, and oxidation state. Thus, correlating these parameters with their final catalytic properties is essential. However, heterogeneity across nanoparticles (NPs) is generally expected. Moreover, their nature can also change during catalytic reactions. Therefore, investigating these catalysts *in situ* at the single-particle level provides insights into how these tunable parameters affect their efficiency. To unravel this question, we applied spectro-microscopy to investigate the thermal reduction of SiO_2_-supported copper oxide NPs in ultrahigh vacuum. Copper was selected since its oxidation state and morphological transformations strongly impact the product selectivity of many catalytic reactions. Here, the evolution of the NPs’ chemical state was monitored *in situ* during annealing and correlated with their morphology *in situ*. A reaction front was observed during the reduction of CuO to Cu_2_O. From the temperature dependence of this front, the activation energy was extracted. Two parameters were found to strongly influence the NP reduction: the initial nanoparticle size and the chemical state of the SiO_2._ substrate. The CuO_*x*_ reduction was found to be completed first on smaller NPs and was also favored over partially reduced SiO_*x*_ regions that resulted from X-ray beam irradiation. This methodology with single-particle level spectro-microscopy resolution provides a way of isolating the influence of diverse morphologic, electronic, and chemical influences on a chemical reaction. The knowledge gained is crucial for the future design of more complex multimetallic catalytic systems.

Oxide-supported metal nanoparticles are excellent catalysts in several chemical production, energy conversion, and pollution prevention industrial processes.^1−3^ Their morphological characteristics, such as size and interparticle distance, and chemical characteristics, such as composition and metal–support interactions, affect the performance of these catalysts.^4^ For instance, supported Au NPs, when smaller than 6 nm, can activate O_2_ in selective oxidation reactions.^5^ The sintering of the NPs, which leads to the loss of active sites and deactivation,^6^ can occur under reaction conditions if the interparticle distance and metal–support interactions are not optimal. Furthermore, how strongly a catalyst binds to the reactants, intermediates, and products depends on the chemical composition and oxidation state of the NPs.^4^ Therefore, isolating and understanding the role of each characteristic of a catalyst is key in order to tune its performance.

Among the different supported metal NPs, copper on silica (SiO_2_) is a system of great interest and broad applicability. For instance, it has an excellent performance in the synthesis of glycols^7−9^ and fuels, such as ethanol.^10,11^ Moreover, this system is also viable for the catalytic conversion of CO_2_ into methanol.^12,13^ One of the focal points of these and other studies is understanding how changes in the oxidation state of Cu-based thermal and electro-catalysts impact their activity, selectivity, and durability. For example, copper oxidation happens during ethylene epoxidation, and the oxidation state of Cu affects the reaction selectivity—CuO produces roughly 10 times more epoxide than aldehyde.^14^ Furthermore, in the direct oxidation of CH_4_ to methanol, it was shown that the reduction rate of Cu-CHA zeolites is highly correlated with their activity, measured as turnover frequency. The reduction of Cu^2+^ is identified as the key step during CH_4_ oxidation.^15^ Similarly, the formation of a Cu_2_O layer increases the reactivity for CO oxidation on Cu(111) catalysts.^16^ In electrocatalysis, this relationship also exists. For instance, in CO_2_ electrocatalytic reduction (CO_2_RR), a passivating copper carbonate layer may form on the surface of cupric-like oxides (Cu^2+^), thus deactivating the dissociative adsorption of CO_2_.^17^ In addition, highly disordered Cu/Cu_2_O/CuO interfaces have been shown to display enhanced selectivity for C_2+_ products in CO_2_RR.^18,19^ Theoretical work from the Goddard group also predicted enhanced C–C coupling when Cu^+^/Cu^0^ interfaces were present during CO_2_RR.^20^ Hence, regulating the catalyst oxidation state is vital for its overall performance.

Moreover, the last step in preparing most supported metal catalysts involves activating them by changing their oxidation state through annealing in a reductive atmosphere.^21^ Therefore, studying the thermal reduction of a supported metal catalyst is of great importance. Regarding copper, a wide range of techniques^22−31^ revealed the mechanisms behind the thermal reduction of its oxides. The thermodynamics and kinetics of this reduction strongly depend on the morphology. Hence, different behaviors for bulk samples,^27^ thin films,^26^ and nanoparticles^31^ are observed. When annealing thin oxide films on thick Cu metal supports in ultrahigh vacuum (UHV) for 30 min, Lee et al. detected that partially oxidized [CuO(1 nm)/Cu_2_O(2 nm)] and fully oxidized [CuO(2 nm)] Cu films required different reduction temperatures to Cu_2_O.^26^ For the former, annealing to only 380 K was necessary, but for the latter, 573 K. On the partially oxidized sample, the reduction mechanism involved oxygen diffusion into bulk Cu, leaving Cu_2_O at the surface. However, this path was blocked on fully oxidized samples, and oxygen preferred reacting with adventitious carbon to form CO. When annealing CuO nanowires in vacuum at 673 K for 2 h, Yuan et al.^28^ detected Cu_2_O NPs decorating the skeleton of the parent oxide, as a result of an incomplete reduction of the original nanostructure. Using *in situ* transmission electron microscopy (TEM), they found that the CuO to Cu_2_O transition happened at the Cu_2_O/CuO interface. Moreover, Cu and O atoms diffused from other regions of the nanowire to this interface for the reaction 2 CuO(solid) → Cu_2_O(solid) + 1/2 O_2_(gas). This reaction, which typically happens above 1073 K upon vacuum annealing, already started at 423 K in their case. Due to the nanostructure morphology, a significant fraction of atoms was available near the surface, thus facilitating the low temperature phase transformation.

Therefore, the reduction of copper oxide species depends not only on the pressure and temperature conditions but also on the structure of the sample and the support used. However, most spectroscopic studies are not combined with microscopy and are based on integral data acquired on large surface areas. Furthermore, the analysis of different sample characteristics and their role in catalysis, such as the size effect on the NP oxidation state, comes from investigations carried out on a series of multiple samples prepared with different methods with distinct average NP sizes that are measured as an ensemble. Conversely, the resulting size dependence has only limited meaningfulness due to the distribution in size, shape, interparticle distance, and other characteristics that are inevitably averaged out when making integral measurements. Therefore, studying changes in individual NPs at the nanoscale is necessary.

Although it is possible to identify the oxide species of the catalyst from the crystal structure in a pure microscopy setup (e.g., lattice parameter in high-resolution TEM),^30,32^ adding spectroscopy measurements enables a more direct and reliable way of gauging a wide variety of species, including those with no or poor crystallinity. In TEM, the standard spectroscopic solution is energy-dispersive X-ray spectroscopy. Nevertheless, this technique is a nonsurface-sensitive averaging method (sampling 1–3 μm of the bulk of the sample),^33^ thus not ideal for catalysis. Combining microscopy and surface spectroscopy can achieve this objective by coupling a specific site with its chemical information. Today’s studies rarely couple these methods to characterize supported catalysts on a nanoscale. Karim et al.^34^ used the surface-sensitive technique near-edge X-ray absorption fine structure (NEXAFS), enabled by an X-ray photoemission electron microscopy (XPEEM), to show that iron NPs with different particle sizes on the same sample, prepared by lithography, had different initial oxidation rates. However, the NP size determination was only possible due to the nature of the nanolithography fabrication. Herein, we apply a similar methodology but on silica-supported Cu NPs, going a step further. By combining low-energy electron microscopy (LEEM) with XPEEM, NEXAFS, and XPS, we gained enough lateral resolution to measure the evolution of the SiO_2_-supported Cu NP size and composition *in situ* during the thermal reduction, while gaining spectral information on specific surface sites. In particular, we were able to detect reaction fronts, and from the temperature dependence of these fronts, we determined the activation energy. These developments indicate that it is possible to use a similar methodology to study complex multimetallic nanostructures under reaction conditions, facilitating thus the elucidation of the role of each individual component on the final catalytic activity.

## Results and Discussion

### Morphology Characterization

**Figure 1:**
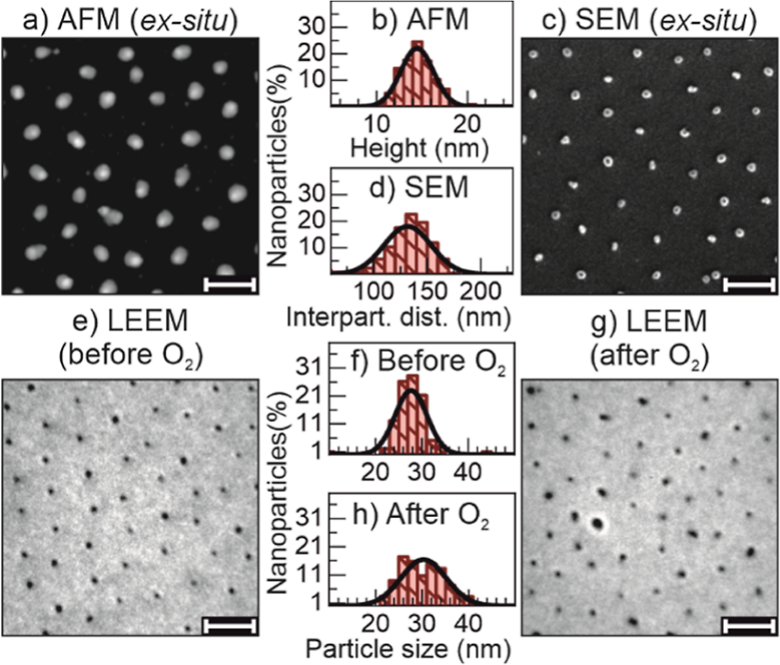
Morphology of Cu NPs supported on Si(100) with a native SiO_2_ layer. AFM (a), SEM (c), and LEEM (e,g) exhibit a homogeneous narrow size distribution and arrangement of the NPs. The NP (b) and interparticle distance (d) distributions are shown. The LEEM data of the pristine (“as inserted in UHV”) sample (e) and after O_2_ annealing (g) show that, both, the NP size and distribution spread (f,h) increased. Both LEEM and SEM images display the same sample but not the same identical area, while the AFM image shows a different, but identically prepared sample from the same micellar solution. The homogeneity of the samples was confirmed on a larger scale (mm); therefore, the displayed images represent the entire surface. The LEEM images were taken at an electron energy of *E*_kin_ = 5 eV. The scale bar is 200 nm long in all cases.

To determine the morphology of Cu NPs supported on SiO_2_/Si(100) (see the Experimental Section for the preparation procedure), we used atomic force microscopy (AFM), scanning electron microscopy (SEM), and after transfer into the UHV chamber, LEEM. Figure 1a shows an AFM image after the *ex situ* O_2_ plasma treatment,^35,36^ which provides accurate height information (Figure 1b), with a mean value of 14.5 nm and a narrow size distribution (95% of the NPs are between 10.9 and 18 nm in size). Figure 1c shows an SEM image of another sample, produced identically in the same batch, also after the *ex situ* O_2_ plasma treatment. Using the SEM data, we estimated an interparticle distance (Figure 1d) of 132 nm (95% of the particles ranging between 87 and 176 nm).

After the *ex situ* characterization, the sample of Figure 1c was transferred into the LEEM/XPEEM UHV chamber. In the LEEM image of the “as inserted in UHV” sample (Figure 1e), it is possible to observe a similar level of detail as in the SEM image. Figure 1e,g shows different areas of the same sample, before and after annealing at 580 K in 2 mbar of oxygen for 2 h. This treatment removed the adventitious carbon and any residual polymer of the sample colloidal synthesis, as proven by a surface-sensitive XPS survey spectrum measured at a photon energy of 400 eV, that shows only peaks corresponding to Si, Cu, and O (Figure S1).

According to previous cross-sectional TEM studies,^36−38^ the NPs synthesized with the method described in the Experimental Section are nearly spherical and have a small contact area with the SiO_2_ support. Therefore, we assumed a circular shape for each NP, calculating their average diameter based on their perimeter determined from the LEEM data (see the Experimental Section). Before the O_2_ annealing, the average NP diameter was (26 ± 3.5) nm (Figure 1f) and was found to slightly increase after the thermal treatment in UHV to 30 ± 5 nm (Figure 1h). The larger NP size measured by LEEM as compared to AFM (NP height, since the diameter is artificially enhanced due to tip effects) is assigned to an instrumental artifact (blurring) of the LEEM measurement due to local distortion of the acceleration field by the NPs. This yields an apparent NP size in LEEM twice the real size as that measured by AFM (NP height, see a comparison of Figure 1b,f and see the Supporting Information for details). The advantage of using LEEM/XPEEM here is that we can monitor not only changes in the sample morphology *in situ* with single-particle resolution but also concurrently the NP’s oxidation state.

### *In Situ* Reduction of Copper NPs

We stepwise annealed the sample (Figure 1g) in UHV. Each step consisted of a fast heating of the sample (approximately 3 min) from room temperature (RT) to a specific temperature, where the temperature was maintained for 10 min, and finally cooled down to RT. Subsequently, after reaching a temperature below 315 K, we investigated the sample state, repeating the analysis after each annealing step at 393, 453, 523, 593, and 643 K. The temperature step size of 60 ± 10 K was chosen to complete the full sample characterization during one synchrotron beamtime. Higher temperatures were avoided to hinder the diffusion of copper through the silica support and to prevent copper silicide formation.^31,38^

**Figure 2:**
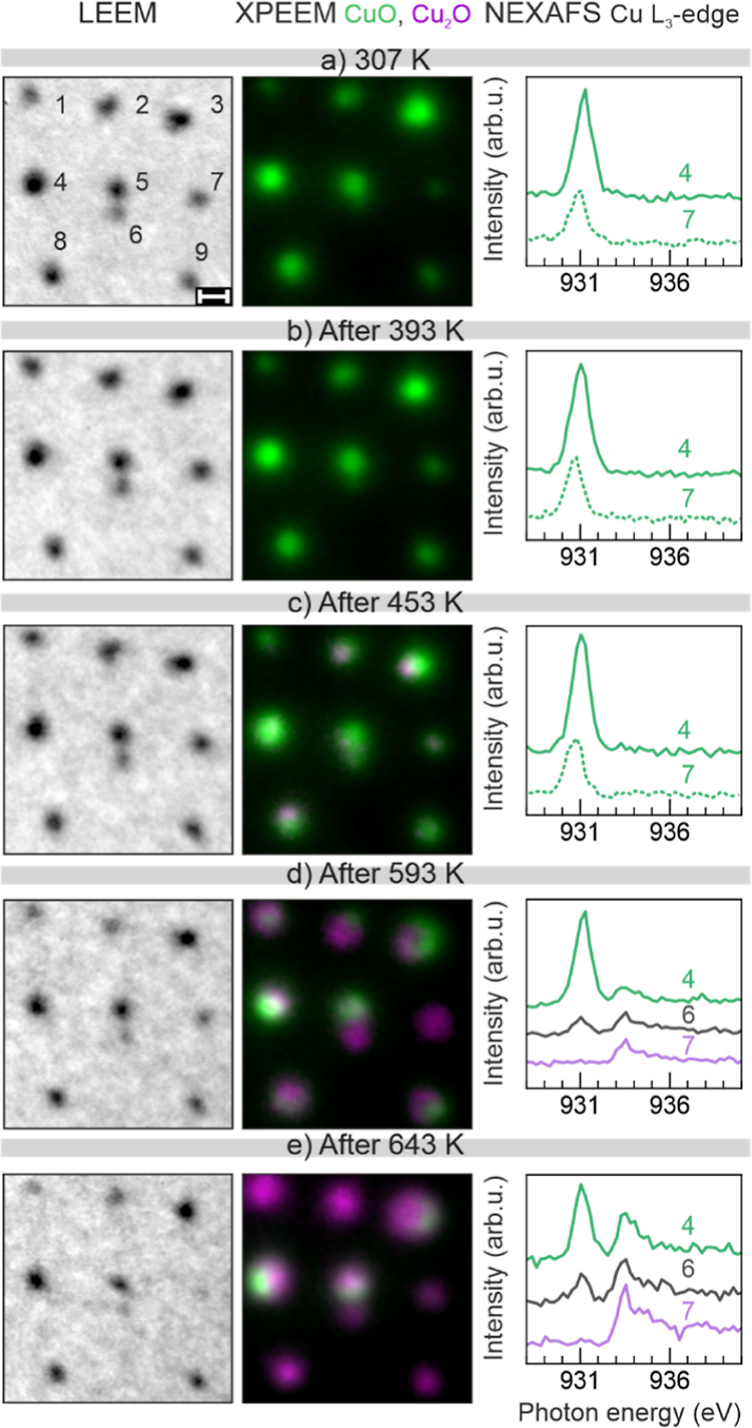
Spectro-microscopy data of Cu NPs after different UHV annealing treatments. Each row contains a LEEM image (left column), a composite XPEEM image of the same area (central column), and local NEXAFS spectra of the Cu L_3_-edge of individually selected NPs. The XPEEM images are both an elemental and oxidation state mapping of the sample: bright areas contain more copper, green-labeled areas correspond to CuO, and magenta to Cu_2_O. All data are taken at RT. While the first row is measured prior to any UHV annealing, the others are measured at RT after annealing the sample at the indicated temperature for 10 min and subsequent sample cooling. After the 593 K annealing, some NPs change significantly, both chemically and morphologically. LEEM images were collected at an electron energy of *E*_kin_ = 5 eV. The NEXAFS spectra are offset vertically for better display. The scale bar in (a) is 50 nm.

By using LEEM and XPEEM (Figure 2) to image the identical NPs, we *in situ* assessed the local changes in morphology and chemical state of the NPs, respectively. Although it is possible to use XPEEM to estimate the sample morphology, LEEM has a higher lateral resolution than XPEEM, enabling more precise measurements.^39^ The reason is that in XPEEM, secondary electrons with a low kinetic energy of *E*_kin_ = 1 eV are used, and these are more affected by the field distortion compared to the LEEM 5 eV electrons. Although the NP size measured in LEEM can be estimated with an accuracy better than 10%, there is a systematic enlargement of the size by a factor of 2 due to local field distortions, as mentioned above. Although the *ex situ* AFM method is capable to determines the real size of the spherical NPs through height measurements, we have used in the present paper the *in situ* method LEEM to estimate the NP size simply because in this way we could follow simultaneously the NP size (LEEM) evolution and its chemical transformation (XPEEM oxidation state). In Figure 2, every image displays the same region with numbered NPs, and the NEXAFS data are created by integrating, at specific sites, a stack of XPEEM images taken at different photon energies (see the Experimental Section).^39^ Moreover, to determine the chemical state of copper, we use the NEXAFS of the Cu L_3_-edge since copper and its oxides have specific and easily discernible fingerprints.^40,41^ In other words, through the NEXAFS (and XPS) data, it is possible to determine not only the oxidation state of copper but also the specific copper species. The CuO species has a strong absorption edge at around 931 eV, while Cu_2_O and Cu^0^ (metallic Cu) have both an intense peak at higher photon energy, 933.6 eV. To further differentiate Cu^0^ from Cu_2_O, one can check for a peak at 937.7 eV, almost as strong as the peak at 933.6 eV, characteristic of metallic copper.^40^ This metallic peak was not observed for any NP, even after the last annealing step. Based on the spectra observed after annealing at 643 K (Figure 2e, right), we selected two XPEEM images to form a composite colored image. One at lower photon energy, assigned to the CuO species, and another at a higher photon energy, Cu_2_O. The images are merged, forming a composite image, in which the CuO XPEEM image is color-coded as green and Cu_2_O as magenta. Therefore, the composite images in Figure 2 are both elemental and chemical state maps, i.e., bright areas contain more copper, and the color indicates the oxidation state. A detailed explanation of the contrast in the composite colored image, and the normalization of the XPEEM images/spectra is provided in the Supporting Information.

Up to 453 K, the LEEM and XPEEM images and the NEXAFS spectra have striking similarities. Within these first three LEEM images (Figure 2a–c), one cannot detect any change in the size, shape, or position of the NPs. In addition, the contrast between the substrate and copper remains relatively the same. Moreover, both the XPEEM mapping and the NEXAFS data show that almost every NP remained CuO, as proven by the spectra of some selected NPs (Figure 2a–c). This oxidation state results from the sample pretreatments, both, the O_2_ plasma etching using in the NP synthesis for ligand removal and the subsequent O_2_ annealing in the mbar range upon introducing the samples in the UHV system. On the other hand, some NPs in the XPEEM composite image (#3, #8) start to show signs of the second phase, with small parts containing Cu_2_O. Regarding the NEXAFS spectra of the NPs, we discuss in the Supporting Information, why some NPs (for example, NP #4) have a higher NEXAFS signal (peak height) than others (NP #7).

After the annealing step at 593 K (Figure 2d), some particles became brighter in LEEM, such as NPs #1 and #7, an effect further intensified by the subsequent annealing (Figure 2e). To understand the cause that led to this contrast change in LEEM, we can resort to the XPEEM and NEXAFS data of the same NPs. In Figure 2d, the spectrum of particle #7 has a distinct peak corresponding to Cu_2_O, showing a complete reduction from CuO. Interestingly, the reduction extent was different for other particles, as shown in the cases of #4 and #6. Moreover, the XPEEM mapping indicates a clear difference in the oxidation state inside the same NP, for instance, in NPs #2 and #3. This observation indicates the presence of a reaction front in each NP, which can start from multiple directions, as evidenced by the left to right progression of particle #3, and from top to bottom in particle #8. Furthermore, the reaction fronts observed are not instrumental artifacts, and they are consistent, as evidenced by the consecutive measurements shown in Figure S11. At a lateral resolution clearly better than 50 nm, the signal overlap of two neighboring NPs, which are on average 132 nm apart (for example, #4 and #6), is negligible. However, at much shorter distances, such as 50 nm as seen for #5 and #6, the signals overlap by about 30%, even though the areas inside the same NP can be clearly discriminated (see also Figure S11). Above 643 K, the particles continued the reduction trend, in which #4 and #6 reduced more, and #7 and #9 already completely reduced to Cu_2_O (Figure 2e). No particle was further reduced to Cu metallic, thus suggesting that either the thermal activation for the complete reduction reaction was not achieved within the temperature range employed, or that the reaction rate was too slow, and thus significantly longer times might be needed.

To reveal why the NPs reduce differently, we employed a statistical analysis, where each NP is an observation associated with different variables, either of morphological (LEEM) or chemical state nature (PEEM/NEXAFS). To establish these correlations, first, we need to reduce the dimensionality of the Cu L_3_-edge NEXAFS data, going from a spectrum to a single variable. As previously discussed, the NEXAFS spectra depict an ongoing reduction, where the NPs go from CuO to Cu_2_O. This reaction can be written as 2 CuO → Cu_2_O + O or Cu^2+^ + e^–^ → Cu^+^. Consequently, we can envision a variable “*f*”, the fraction of Cu^2+^ converted into Cu^+^. One way of deriving this variable is to use the peak intensities (amplitudes) to quantify the amount of each species in an NP. This is easily possible because the Cu L_3_-edge NEXAFS spectra of both CuO and Cu_2_O are characterized by single absorption peaks at 931 eV for Cu^2+^ and 933.6 eV for Cu^+^, and the intensity of these peaks are proportional to the number of atoms of these species in a NP. Therefore, the equation for the converted fraction based on the experimental intensities can be written as
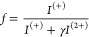
1

Whereas γ is a weighting factor that compensates for the use of peak intensities rather than their integral intensity, as well as for the differing cross sections of the two species. It is experimentally determined as γ = 0.84. A detailed and comprehensive explanation of how we derived both the equation and the weighting factor can be found in the Supporting Information. This γ value might be temperature-dependent; however, we measured all NEXAFS data at RT and therefore, we can rule out any temperature effects. Now, for each NP, and annealing step, there is a *f* value derived from the NEXAFS (chemical) data. The following sections discuss how the morphological variables of the NPs correlate with the *f* values, and how the chemical state of the substrate influences *f*.

### NP Morphology and Oxidation State

**Figure 3:**
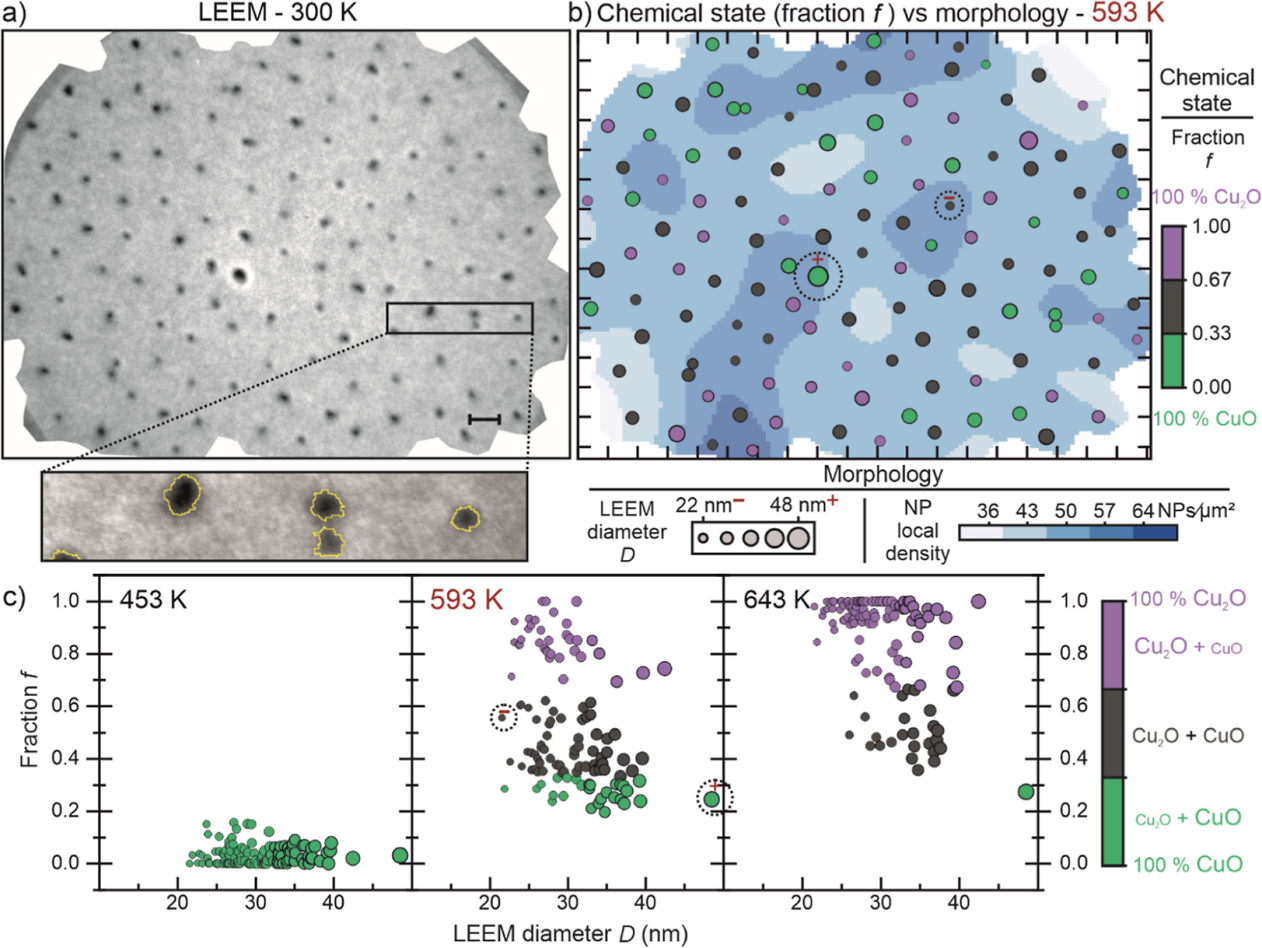
Morphology effect on the reduction of NPs. (a) LEEM image before annealing in UHV is used to extract the morphological information, which is then combined with the NEXAFS data. There are two different color-codings in image (b), one for the background and another for the NPs. The first has a blue color scale, where regions with a low density of NPs are bright blue, and those with a high density of NPs are dark blue. While the NPs, represented by circles scaled by the size obtained in the LEEM image, have colors based on the converted fraction *f* to Cu_2_O after the 593 K annealing step. When *f* is between 0.67 and 1.0, the NP color is magenta, mostly Cu_2_O. Dark gray NPs have *f* between 0.33 and 0.67, and are a combination of CuO and Cu_2_O. Finally, green NPs have more CuO, having an *f* ratio ranging from 0 to 0.33. (c) For different annealing temperatures, the progression of the converted fraction *f* against the NP diameter *D* measured in LEEM. The scale bar is 100 nm.

Several morphological features can influence how a nanostructure reduces or oxidizes. The possible factors for the different reduction patterns observed must be variables with high enough variance in the population. Factors such as shape and crystallinity (no LEED patterns), while they definitely can impact the redox capacity, cannot explain the different *f* values of the NPs because of their uniformity in our sample. Conversely, two heterogeneous features that can be reliably extracted from LEEM are the size and position of the NPs. The size of the NPs is a straightforward variable, represented by their diameter, considering spherical NPs. We extract this information from the NP perimeter (Figure 3a insert). The position should be determined relative to other sample characteristics, such as substrate features or other NPs. Since the substrate is relatively homogeneous, containing no distinctive features or roughness in the LEEM image (Figure 3a), we focused on calculating the interparticle distance. The local density and the nearest neighbor distance are two variables that can affect chemical reaction dynamics. The former describes the agglomeration of NPs, and the latter describes the proximity of a NP to its closest NP. In case of thermally induced NP sintering, the size of the resulting NPs will increase, which is expected to influence their reducibility. The explanation of how these variables are calculated is included in the Experimental Section, and a discussion on how these factors can impact the course of a chemical reaction can be found in the Supporting Information.

Now that we have the morphological variables and *f* values for each annealing treatment, we can group these data in a map to detect visible patterns. Figure 3b shows the state of the sample after the 593 K annealing step. Same-colored NPs (similar *f*) are not grouped in any discernible fashion, although smaller NPs are predominantly magenta-colored, thus Cu_2_O. To observe if this statement holds through for the different annealing steps, we plotted in Figure 3c the *f* ratio versus the LEEM diameter *D*. A ratio of *f* = 0 indicates a fully oxidized NP, while a ratio of *f* = 1 means a NP fully reduced to Cu_2_O. The 593 and 643 K graphs show that smaller NPs reduced first from CuO to Cu_2_O.

To properly quantify the correlations between the morphological variables and *f*, we used Spearman’s rank correlation coefficients.^42^ This coefficient is the Pearson correlation coefficient between the rank variables. The raw data, such as the NP LEEM diameter *D* (as an example, the arbitrary values 25, 30, and 28 nm), are converted into ranks (first, third, and second). Therefore, it is possible to monotonically correlate variables that do not necessarily have a linear relationship, which is the case here. A perfect correlation has a value of +1 or −1, while a correlation of 0 indicates no tendency for one variable to either increase or decrease when the other variable increases. As Figures 3c and S7, depict, the NPs did not reduce in the initial temperatures. However, after the 593, and 643 K annealing steps, the reaction progressed enough to identify correlations. For the first one, the correlation coefficients for nearest neighbor distance, local density, and NP size were 0.06, −0.08, and −0.41. For 643 K, the values were 0.04, −0.22, and −0.43. Hence, the first two variables correlate weakly with *f*, and the third, albeit not a strong correlation, is worth a more careful examination. One of the reasons for a not-so-strong correlation would be a not-so-perfect determination of the size of the nanoparticles, since this characteristic is estimated from the pixel intensities to detect the perimeter. Speaking of Spearman’s rank correlation, it could be that the ranking fails in a small range of data. For example, two nanoparticles with 25 and 26 nm LEEM diameters may have, in reality, 26 and 24 nm. Nevertheless, this estimation error does not affect the NPs with a considerable difference in size. A way to mitigate this issue is to group the NPs in size bins. The sizes and number of NPs in each bin are described in Table S2. Due to the inelastic mean free path length (IMFP) of about 3 nm for the detected secondary electrons, the information depth, from which 95% of the signal comes from, is *L* = 3 × IMFP = 9 nm. Therefore, one can determine the oxidation state only from the outer portions of the NPs. One can assume that the particles of the same size have all the same oxidation state, meaning a fraction *f* of the NP is reduced, and the rest is still fully oxidized. However, the NPs in our sample might be rotated against each other (Figures S12 and S13). Therefore, some are reduced on top (fully visible), some in the bottom (not visible), and some at the side (partially visible). This leads to a scattering of the data for one NP size, as shown in Figure 3c. However, when averaging these data, a meaningful value is created. In fact, this averaged value describes the oxidation state only in the outer shell with a thickness of *L* = 9 nm which is enough for NP sizes of up to 2 × *L* = 18 nm as in our case, as determined by AFM (see Figure 1b). However, for larger particles, the inner core is inaccessible with this method. The physical meaning of the data binning is explained in depth in the Supporting Information, which explores the effects of multidirectional reaction fronts and information depth on the experimental signal (Figures S12–S15). Grouping highly increases the Pearson correlation between *f* and *D* for the 593 K data from −0.41 to −0.8 and for the 643 K data from −0.43 to −0.95.

**Figure 4:**
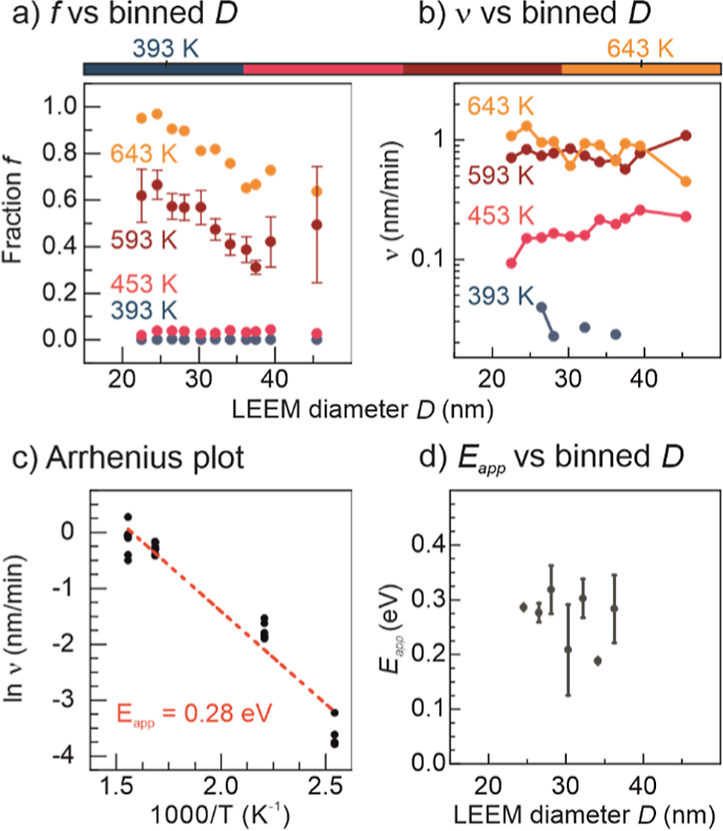
Apparent activation energy. (a) Average *f* ratios for NPs binned by their size. (b) Front velocity *v* dependency with the diameter of the NPs for each temperature step. (c) Apparent activation energy calculated from an Arrhenius plot, where only bins with enough NPs to be statistically sound are plotted (as points). The standard error for the 593 K red curve in (a) is presented. For the other curves, the standard error is ±10%, and the complete Figure 4a with error bars is provided in Figure S19.

Figure 4a clearly displays this relationship. Therefore, size strongly predicts the reaction completion for a given NP, with smaller NPs fully converting into Cu_2_O first than larger ones. This phenomenon can be interpreted either by the chemical reaction being faster in smaller NPs, or just a matter of geometry, where a smaller volume needs less time to be converted to Cu_2_O than a bigger one. We calculated the reaction front velocity for the different-sized NPs to identify the correct interpretation. The equation for the temperature-dependent velocity *v* is
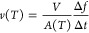
where *V* is the volume of the NP; *v* and *A* are the temperature-dependent (*T*) front velocity, and front area, respectively. Δ*t* is the time for each annealing treatment (10 min), and *f* is the aforementioned converted fraction of CuO into Cu_2_O, whereas Δ*f* is the change of *f* in respect to the previous annealing step. How this equation is derived and why the area of the reaction front changes depending on the temperature (more precisely *f*) are described in the Supporting Information. Figure 4b shows that the front propagates faster when the temperature increases. However, as the horizontal curves show, the NP reduction front moves at similar speeds for every size, signifying that the size dependence is related to the volume to be converted and not to a difference in active sites of smaller NPs (for mechanisms responsible for size-dependent reactivity, see the discussion in Roldan).^4^ The larger the NP, the bigger the volume that needs to be converted/reduced; thus, it will take longer to reach the complete conversion. One important remark is that for one of the annealing steps (523 K), it was not possible measuring PEEM/NEXAFS of the NPs and thus, a correction was applied, which is explained in the Supporting Information.

Moreover, from the front velocity *v*, it is possible to calculate the apparent activation energy (*E*_app_) of the reduction reaction, through the formula
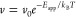


The derivation of this equation is exposed in the Supporting Information. By displaying the front velocity in an Arrhenius plot [i.e., In (*v*) versus 1/*T*], we can directly retrieve *E*_app_ from the slope of the curve. Kim et al.^22^ calculated an apparent activation energy *E*_app_ for the H_2_ reduction of CuO to Cu^0^ of about 14.5 kcal/mol (or 0.63 eV), while the value is 27.4 kcal/mol (1.19 eV) for Cu_2_O to Cu^0^, both on powder samples. Fedorov et al.^43^ found an *E*_app_ of 38 kJ/mol (0.39 eV) for a CuO catalyst reduction by H_2_ to Cu^0^, while Li and Mayer^44^ reported an *E*_app_ of 1.1 eV for the CuO thin film reduction in vacuum to Cu_2_O. Our apparent activation energy *E*_app_ for the reduction of CuO to Cu_2_O for silica-supported NPs in UHV of 0.28 eV is smaller than the previously reported values in the literature. This might be assigned to the following reasons: (i) the CuO reduction took place in our case in UHV under very clean conditions (10^–10^ mbar pressure range) and not in H_2_ pressure which can alter the reaction pathway, (ii) the average NP size is different in our samples from those in prior works, and since the reduction appears to be size-dependent, different values for *E*_app_ are expected when comparing with thin films in the literature or in average, differently sized NPs, and (iii) the NP support selected and the density of defects within the support and the NPs might also affect *E*_app_. Interestingly, the smaller activation energy might explain why the NPs reduced first to Cu_2_O, and not directly to Cu^0^.

### Substrate Chemical State Influence on the NP Oxidation State

**Figure 5:**
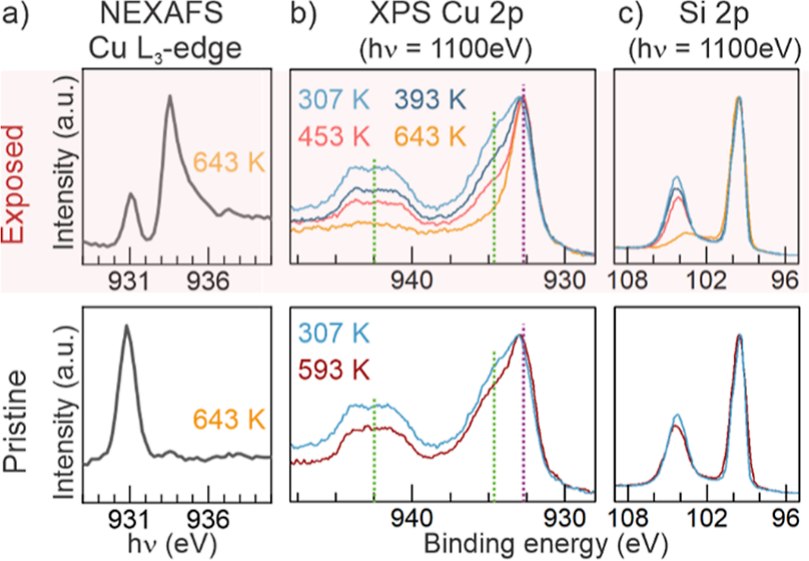
Chemical state changes in two different types of SiO_2_ areas with distinct amounts of X-ray exposure. In the exposed regions, top row, multiple measurements are performed before each annealing step, while in the pristine areas, bottom row, the measurement is performed only after one annealing step. (a) NEXAFS of the Cu L_3_-edge and (b) XPS of the Cu 2p showing reduction of the NPs on the pre-exposed SiO_2_ areas and CuO on pristine SiO_2_. (c) XPS spectra of the Si 2p showing that the substrate is significantly reduced in the exposed areas. NEXAFS and XPS were measured in different zones to mitigate beam damage.

Although the NP size had a high correlation with the NP’s oxidation state (*f*), we noticed another pattern when measuring in different areas, tens of μm away from the one in Figure 3a. There, the NPs were still CuO. To understand what underlying factor was responsible for hindering the reduction of NPs in some areas or perhaps enabling it in others, we measured NEXAFS and XPS in two different types of areas. The first type, denominated “exposed areas” (Figure 5a, top), corresponds to areas where consecutive X-ray measurements (which will be referred to as X-ray exposure) were performed after each annealing step. In contrast, the second type, “pristine areas” (Figure 5a, bottom), had no X-ray exposure before the annealing steps. In summation, the exposed areas had two significant differences from the pristine ones, a higher X-ray total exposure and a different order of processes: X-ray exposure → annealing → X-ray exposure, while the “pristine areas” were exposed to annealing → X-ray exposure (during measurement). The spectra recorded in these two different types of regions are entirely different: while in Figure 5a, in the exposed area, every particle initiated the reduction process to Cu_2_O, and 64% of the NPs were entirely in the Cu_2_O-oxidation state; in the pristine areas, no NP had changed its oxidation state, even after the same annealing step, at 643 K.

The XPS spectra in Figure 5b tell a similar story: the Cu 2p peak continually shifts to lower binding energies during annealing, transitioning from having a bigger CuO component to an almost single Cu_2_O one.^45^ Moreover, the Cu_2_O component is detected even at lower annealing temperatures, such as 393 K (Figure 5b). This reduction was not detected in the NEXAFS spectra at the same temperature (Figure 2), in which no Cu_2_O component was found. However, there is a difference in the sampling depth of each technique. The XPS measurements, performed with a photon energy of 1100 eV, are more surface sensitive than the NEXAFS because the detected Cu 2p photoelectrons have around 160 eV of kinetic energy, which can be translated to an inelastic mean free path (IMFP) of only 0.55 nm.^46^ On the other hand, in our NEXAFS experiments, we detect secondary electrons, which have up to 5 nm of IMFP.^47,48^ Therefore, the different results of both techniques could be explained by the disparity in the IMFP of the detected electrons of each technique. A reduction that starts from the top and goes to the bottom, would be detected first, or more easily by the most surface sensitive technique (XPS).

Despite the expected irreducibility of the SiO_2_^49^ thin support layer, the XPS spectra of Figure 5c (top) surprisingly show successive silica reduction in the exposed areas. Therefore, Figure 5 unveils a simultaneous reduction of both, NPs and the substrate. Before the annealing steps, the characteristic peaks of SiO_2_ and Si^0^ are prominent. However, as soon as the first annealing step was performed, the oxide peak decreased and shifted to lower energies, intensifying with higher annealing temperatures and more X-ray exposure. It is important to clarify that we normalized the spectra by the Si^0^ component at 99.4 eV to facilitate peak comparison. Although, both peaks changed their heights. Explicitly, the oxide decreases as the Si^0^ increases. After 643 K, the silicon oxide peak is barely present. However, the SiO_2_ sublimation temperature is above 1373 K in UHV. Even though metallic NPs can catalyze the SiO_2_ decomposition, lowering the required temperature, a different fingerprint is left behind, with the formation of pores and ridges. Ono and Roldan Cuenya^58^ showed an example of this phenomenon: Au NPs accelerated the desorption of oxygen and decomposition of SiO_2_ underneath and around the NPs in UHV at 1000 K. However, we did not observe these phenomena. Instead, we detected an almost complete silicon oxide removal at significantly lower annealing temperatures. On the other hand, the substrate was barely reduced in the pristine areas, even after annealing at 593 K, signifying that this reduction process could not be only thermally mediated and that it could not be only explained by the catalytic role of the surrounding NPs. Similarly, to the Si 2p spectra, the Cu 2p spectra of the pristine areas show small variation between the RT and 593 K curves, indicating a correlation between the reduction of the NPs and the substrate.

**Figure 6:**
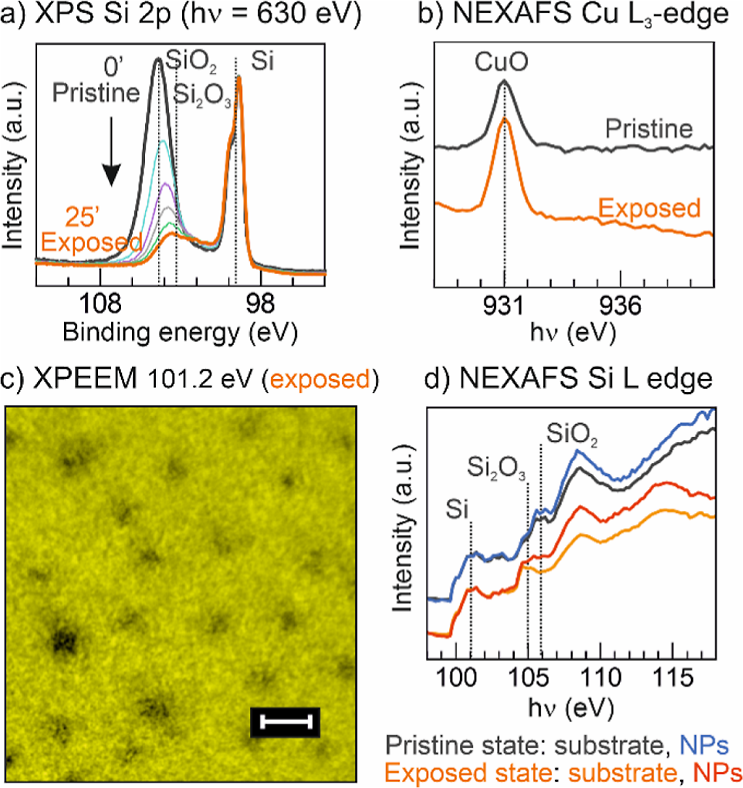
Effect of X-ray exposure tested at RT. (a) During the XPS measurement of the Si 2p core level, with a high flux of X-rays, the silicon oxide layer is reduced, both in thickness and oxidation state. (b) NEXAFS of the Cu L_3_-edge shows that the NPs do not reduce with X-ray exposure alone at RT. (c) XPEEM at *h*ν = 101.2 eV of the exposed state and (d) local NEXAFS of the Si L-edge of both pristine and exposed state. For each state, spectra were recorded for two different regions separated based on the pixel intensity at the XPEEM image at *h*ν = 101.2 eV: the substrate, and the NPs. The former corresponds to the continuous yellow area of (c), in which no NPs are present, and the latter corresponds to the darker pixels in the XPEEM image, which have NPs in their center. The pristine state has both, NPs and substrate regions with almost the same fingerprint (Si^0^ and SiO_2_ signals), while in the exposed state, the NPs and substrate have two new fingerprints. The NPs region has the fingerprints of Si^0^, Si_2_O_3_, and SiO_2_, while the substrate region has only Si^0^ and Si_2_O_3_. The NEXAFS spectra were subtracted by the pre-edge and normalized by the peak intensity of the Si^0^ component at *h*ν = 101.2 eV. Moreover, we applied an offset to separate the pristine spectra from the exposed state ones. The scale bar is 100 nm long.

To further understand the substrate reduction and decouple the effect of the X-ray exposure from the thermal treatments, we performed a second set of measurements on another similar sample. This sample, however, did not have any prior or subsequent thermal treatment. For instance, to remove the adventitious carbon from this sample, we resorted only to *ex situ* and *in situ* oxygen plasma treatments at RT. The experiment consisted in continuously measuring the Si 2p core level (Figure 6a), but instead of using the regular X-ray dosing, we increased it 10-fold, anticipating the acceleration of the substrate reduction. At a photon energy of 630 eV (the selected photon energy for this monitoring), the IMFP for the Si 2p core level is 1.5 nm. Considering a total information depth of three times^50^ the IMFP, the photoelectrons come both from the oxide layer (which has a thickness of around 2 nm for native oxides)^51^ and the substrate underneath (Si^0^). As soon as the exposure started, the oxide peak started to decrease and shift to lower energies, as evidenced by the descending colored curves taken at 5 min intervals. At the end of 25 min, the sample was stable, and the orange curve shows that the main oxide component shifted 1 eV to lower binding energies. This shift makes Si_2_O_3_ the main oxide species after the prolonged X-ray exposure.^52^ Typically for native silicon oxides on a silicon wafer, an interface exists with Si atoms in an intermediate oxidation state, SiO, Si_2_O, and in a more considerable amount, Si_2_O_3_.^53^ Furthermore, considering the final heights of both Si_2_O_3_ and Si^0^ peaks, the remaining oxide species are present in a significantly lower amount than the original SiO_*x*_.

Strikingly, the oxide peak shift and decrease in height are the same as those previously observed in the thermal annealing experiment (Figure 5c). Therefore, this trend is associated with the X-ray exposure independently of the thermal treatments. Hence, in the exposed area of Figure 5, the substrate was in a more reduced state before each annealing step, and therefore, this might be why the NPs preferably reduced just in these areas. It is relevant to clarify that we also tested, in other regions, the exact X-ray dosage used for the thermal annealing experiment (Figure 2), and the result was similar. Albeit slower, the X-rays still reduced the SiO_2_ layer. Interestingly, dosing O_2_ in the 10^–7^ mbar range simultaneous to the X-ray dosage (in other words, providing an oxygen reservoir) halted the silica reduction (see Figure S19 in the Supporting Information).

X-ray-induced changes in silica are a well-studied phenomenon since different applications which depend on SiO_*x*_ materials suffer from its reduction due to the radiation exposure.^54^ One of the most common radiation-induced effects is the formation of electron–hole pairs and, consequently, point defects. One of the most frequent is the E′ center, in which a rupture of a Si–O bond leads to an unpaired electron in a Si dangling bond. Another is an oxygen-deficient center, where an energetic photon (UV and X-ray) releases an interstitial oxygen atom from the silica, resulting in a ≡Si–Si≡ covalent bond^55,56^ (three different Si–O bonds). This process differs from those in which oxygen is removed from reducible oxides, where excess electrons are redistributed on the cation empty levels.^57^ What we observe here for the SiO_2_ agrees with the literature: removal of oxygen from the lattice without a significant increase in the Si^3+^ species, as observed in the XPS spectra dominated by Si–Si bonds. Although the substrate changed significantly during X-ray exposure, the NPs did not. Given that the Cu L_3_-edge NEXAFS spectrum, in Figure 6b, shows no sign of the Cu_2_O species, it is evident that thermal activation by annealing is also necessary to reduce the nanoparticles.

To gain further insights into how the reduction of the substrate happens on a local scale, we measured XPEEM, Figure 6c, and NEXAFS of the Si L-edge, Figure 6d. In the XPEEM image in Figure 6c, taken at the Si^0^ NEXAFS peak energy, the nanoparticles are darker than the substrate, indicating that they have less silicon than the substrate. However, the Si signal is not absent. Given resolution limitations and a sample containing spherical objects, some Si signal from the sides is expected (Figure S5). Alternatively, silicon or silica may also encapsulate the NPs, but probably only partially, since the sample was not annealed, thus limiting the diffusion. Furthermore, a strong Cu 2p XPS signal (Figure 5b) at a surface-sensitive kinetic energy (IMPF = 0.55 nm) contradicts the encapsulation of the Cu NPs with multiple layers of Si or SiO_*x*_.

Figure 6d shows that in the pristine sample, the NPs (black curve) and the substrate (blue curve) show similar Si L-edge NEXAFS curves containing peaks corresponding to Si^0^ and SiO_2_. On the other hand, in the exposed state curves (red and orange), the SiO_2_-related peaks at 106 eV decrease, and the Si_2_O_3_ ones at 105 eV, previously a shoulder in the pristine curves, become more evident. This behavior supports the XPS observations of the same species (Figure 6a).

Unexpectedly, the NPs’ region has a different composition than the substrate after X-ray exposure. Although both, NPs and substrate regions contain signs of Si^0^ and Si_2_O_3_, only the red NP curve has peaks related to SiO_2_. Before drawing conclusions, it is important to state that the Si NEXAFS signal is significantly smaller in the NP region (Figure S5), even more than in the pristine state. In brief, both regions, with and without NPs, were affected by the X-ray exposure, but the SiO_*x*_ in close proximity to the NPs has a different composition than the SiO_*x*_ apart from the NPs. One could assume that the NPs form a protective barrier against the X-rays, decreasing the amount of damage on the substrate just below the NPs. However, the X-rays penetration depth is in the μm range, while the particles here are in the nanoscale. Thus, every area illuminated by the X-rays is affected. Another possibility is that this different fingerprint after X-ray exposure is a sign of a specific interaction of the SiO_*x*_ layer in close contact with the metal NPs. A reduced oxide substrate (Si^3+^) and NPs (Cu^2+^) in the immediate vicinity of the support at a higher oxidation state (Si^4+^) could indicate electron transfer between the metal and the oxide.

Therefore, we have shown that X-ray exposure facilitates the reduction of the nanoparticles, but not directly. The reduction of the CuO nanoparticles is a two-step process, starting with a local X-ray-induced reduction of the substrate and followed by the removal of oxygen from the CuO lattice through thermal annealing, favoring oxygen spillover from the oxidized Cu NPs to the partially reduced SiO_*x*_ support. The thermal activation of the diffusion– either on the surface or through the bulk of the NPs– explains the observed temperature dependence. In the case of the nonexposed area, the supporting SiO_2_ film is not reduced, thus it does not promote the reduction of CuO by spillover. This spillover mechanism is commonly found in catalysts that have a reducible substrate. Ono and Roldan Cuenya^58^ showed that gold nanoparticles could reduce at significantly lower temperatures (300 K) when deposited on TiO_2_*vs* on SiO_2_ (500 K). There, the proposed mechanism involved the spillover of atomic oxygen from the NP shell to the substrate, replenishing the oxygen vacancies created on TiO_2_ upon annealing. Considering that, in our case, the normally nonreducible silica oxide was already prereduced by the X-ray exposure, this pathway becomes a possibility also for the SiO_*x*_ support. Regarding copper systems, a parallel can be traced between our scenario and the one observed when thin copper oxide films on copper bulk crystals are annealed in UHV. In this case, the diffusion of oxygen atoms into the bulk happens, and the CuO thermal reduction to Cu_2_O occurs at a much lower temperature, 573 K,^22^ than the one observed in bulk crystals, 1073 K^59^ (A less-likely alternative mechanism is presented in the Supporting Information). Furthermore, the X-ray-induced reduction of the Si oxide support, which in turn facilitates the reduction of Cu oxide, could impact the catalytic performance of Cu-based catalysts. In chemical processes where metallic Cu or partially reduced Cu oxides serve as catalysts, this could lead to an enhanced reactivity, but there is also the risk of forming Cu silicide species,^31^ which can affect the catalytic performance.

Ultimately, our findings and methodology have several implications for the broader field of catalysis. These include the possibility of fabricating heterogeneous catalysts, for instance, with two distinct crystalline and chemical phases within the same nanoparticle, where each phase may be responsible for a different reaction step. This is feasible since two neighboring sites with different oxidation states can react or adsorb differently various complexes. For example, the coexistence of Cu^+^ and Cu^0^ has been reported to enhance the selectivity toward C_2+_ hydrocarbons during the electrocatalytic reduction of CO_2_.^18,20,60^ Moreover, highly disordered Cu^+^/Cu^2+^ interfaces were also found to favor the formation of oxygenates, in particular ethanol.^19^

Our work also shows that it is possible, through a combination of X-ray exposure and annealing at lower temperatures, to engineer the substrate and/or the NPs locally (on a μm-scale), without affecting the rest of the sample. This technique can thus serve to generate defects that can be placed at desired surface regions, which might be used as a preparatory “activation” step before a chemical reaction.

Moreover, for heterogeneous samples, it enables the study of particle size and particle shape effects within a single sample, since the structural and chemical evolution of numerous individual particles can be resolved. Moreover, different catalytic materials or distinct promoter species available within one sample could be studied under identical reaction conditions (pressure, temperature, and applied potential), as long as they are spatially distributed further away than the resolution limits of our spectro-microscope, namely, 18 nm in XPEEM^61^ and 2.6 nm in LEEM.^61^ The same applies to bi- and multimetallic nanoparticles, whose chemical transformation during catalysis could be spectroscopically monitored not as an average of all particles within a sample, but as a function of the individual composition of each NP. However, the great challenge that remains here is the development of highly sensitive and localized experimental detection methods that would allow to extract reactivity information from a single particle, which is normally not achievable with conventional mass spectroscopy or gas chromatography methods due to the low product yield.

In our specific example, we identified that under identical reaction conditions, smaller NPs reduce first, a distinction that an integral approach could not achieve, as it would result in an average of every characteristic across the entire population. Thus, this method allows the study of multiple parameters in a single experiment or reaction and ensures reliability since the identical pressure or temperature conditions are guaranteed.

## Conclusions

Using a methodology that involves spectro-microscopy (LEEM/XPEEM) and statistical analysis, we successfully correlated the morphological properties of size-controlled Cu NPs supported on SiO_2_ with the oxidation state of both, the Cu NPs and the SiO_*x*_ substrate. Moreover, we were able to *in situ* detect different oxidation states within the same NP during UHV annealing. The reduction mechanism of the NPs was elucidated, where a single-step reduction from CuO to Cu_2_O happens without the formation of metallic Cu up to 650 K. The particle size was the NP characteristic that affected the most the reduction of the NPs. Also, we detected the presence of reaction fronts, calculating their speed, and the apparent activation energy of *E*_app_ = 0.28 eV for the reduction of CuO to Cu_2_O on SiO_2_-supported NPs. Moreover, the reduction of the silica substrate through X-ray exposure was found to be necessary to start the CuO reduction at temperatures lower than 643 K, in this case at 453 K. The ability to produce oxygen vacancies *in situ* at localized sites, as demonstrated here via synchrotron radiation, can be invaluable when studying the impact of oxygen vacancies on catalytic reactions, since they can not only act as spillover sites but also enhance inert molecule adsorption. Furthermore, the single nanoparticle spectro-microscopy methodology demonstrated here could be expanded to the study of multimetallic catalysts under working conditions. Moreover, this work contributes to the ongoing discussion on the importance of studying the chemical state of the support in parallel to the active catalyst, even for metal oxides considered irreducible since external environmental factors, including the use of noninnocent experimental probes might strongly affect the physicochemical phenomena under study.

## Experimental Section

### Nanoparticle Synthesis

Using the inverse micelle encapsulation technique,^35^ we produced copper NPs. Their size and interparticle distance^36^ was controlled using a suitable deblock copolymer, PS(248,000)-P2VP(195,000) from Polymer Source Inc. To deposit the NPs on clean oxidized Si(100) substrates (10 mm × 10 mm), we dip-coated the substrates in a solution containing the copper-filled micelles. Finally, *ex situ* oxygen plasma (30 min, 0.48 mbar) removed the micellar polymer. At this stage, only copper nanoparticles remained on top of a native silica layer; however, some adventitious carbon, after subsequent air exposure, is present.

### Experimental Setup

The *in situ* experiments were performed in a LEEM/XPEEM microscope (SMART) in the low 10^–10^ mbar range, operating at the UE49PGM undulator beamline of the BESSY II synchrotron light source at the Helmholtz Center Berlin (HZB). The aberration-corrected and energy-filtered LEEM-XPEEM system achieves a lateral resolution of 2.6 nm in LEEM mode.^61^ AFM images were acquired in taping-mode with a Digital Instruments Nanoscope III microscope, and SEM images were taken with a Thermo Fischer Scientific Apreo SEM.

### Nanoparticle Height, Interparticle Distance, and Diameter Calculation

Using a local threshold algorithm in a 16-bit image of the Fiji software,^62^ we created two regions, one continuous region, the background, and another of NPs. “Local” means here that the threshold is computed for each pixel based on the intensity of pixels inside a specified radius. If the pixel is above the average of the local maximum and minimum, it becomes white; otherwise black. This method is advantageous in comparison to global thresholds (over the whole image) in cases where the background intensity is heterogeneous and possible background corrections are not reliable. Working with the 16-bit image of the raw data prevents us from compressing the data and losing information when thresholding. Using the analyze particles algorithm of Fiji, we created a region of interest (ROI) for each NP. The perimeter of this region was used to estimate the LEEM diameter of each NP. For Figure 1, we used the SEM image as the base for the coordinate extraction, while for Figure 3, we used the LEEM image. With the coordinates, we used a kernel algorithm of spatstat^63^ as a R package, to calculate both the nearest neighbor distance (1st order), and the NPs local density in different locations. This algorithm produces a smoother density map that also considers irregular boundaries, such as the grayscale area delimited by the white space, in Figure 3a. The height was extracted from the AFM data, using the same threshold mechanism, and taking the maximum height of each ROI.

### NEXAFS Extraction from PEEM Data

The XPEEM system achieves a lateral resolution of 18 nm,^61^ and the overall energy resolution was in the range of 200–750 meV for the NEXAFS measurements, and for the XPS measurements, between 500 and 800 meV. Two different ways of collecting XPEEM images were applied: (1) varying the kinetic energy of the detected electrons, similarly to XPS, while keeping the photon energy fixed, or (2) vice versa, varying the photon energy while fixing the detected electron energy. By varying the X-ray energies across an absorption edge, one collects near-edge X-ray absorption spectroscopy (NEXAFS) spectra, whereas Auger or secondary electrons are detected. While the first option is surface sensitive, often less than 1 nm, the second is more bulk sensitive (2–10 nm). At low energies, the secondary electrons have a high emissivity, and the microscope has the highest transmission, making the secondary electron detection mode much more intense than the Auger electron one and, therefore, the optimal choice. Collecting a reliable NEXAFS spectrum for each NP demands the following condition to be fulfilled: a high-intensity signal coming from a nanoscale area (30 nm^2^), about 1.6 × 10^6^ times smaller than the overall irradiated X-ray area (48 μm^2^). Another particularity in these measurements is the energy selection of specific secondary electrons, with a physical slit that only accepts photoelectrons within a 0.5 eV range, optimizing the contrast between substrate and NPs, namely, partial electron yield NEXAFS. To generate the NEXAFS spectra for the same NP across different photon energies and annealing temperatures, we aligned every XPEEM image with each other to compensates for image drifts. Finally, we extracted the Cu L_3_-edge NEXAFS spectra of more than 100 nanoparticles across the different annealing steps. Each point in the NEXAFS spectrum of a single NP is defined as the average of every pixel inside the NPs perimeter at a given energy (this technique also improves the averaged NEXAFS signal after each annealing step, displayed in Figure S7, since we are removing the contribution of the substrate pixels to the overall spectra). A comparison with complementary spectroscopic electron microscopes is given in Table S3 in the Supporting Information.

## Supporting Information Available

XPS survey of the sample after the initial O_2_ annealing treatment; LEEM blurring effect explained; XPEEM composite image—color and contrast stretching; correlation between the NP size and NEXAFS peak intensity; normalization of XPEEM images and spectra; NEXAFS data expressed as a *f* variable and the importance of weighting factor; relevance of the position of NPs; data binning, front direction, and NEXAFS simulation; equation for the temperature-dependent front velocity; front area changes due to geometry, diameter, and*f*; correction of missing measurement of the annealing step 523 K; Arrhenius plot and apparent activation energy; alternative reduction mechanism; compensation of beam damage by oxygen dosage; and comparison with other spectro-microscopes (PDF)
