# Supplementary material for: Correlative In Situ Spectro-Microscopy of Supported Single CuO Nanoparticles: Unveiling the Relationships between Morphology and Chemical State during Thermal Reduction
Source: ACS Nano. 2024 May 14;18(21):13714–25. doi: 10.1021/acsnano.4c01460 (PMC11140838; doi:10.1021/acsnano.4c01460)
Supplement: Supplementary file 1 — nn4c01460_si_001.pdf [file nn4c01460_si_001.pdf]

Correlative In Situ Spectro-Microscopy of Supported Single CuO Nanoparticles: Unveiling the Relationships between Morphology and Chemical State during Thermal Reduction

Lucas de Souza Caldas, Mauricio J. Prieto, Liviu C. Tănase, Aarti Tiwari, Thomas Schmidt* and Beatriz Roldan Cuenya*

Department of Interface Science, Fritz-Haber Institute of the Max-Planck Society, Berlin 14195, Germany.

Supporting Information

Morphology characterization

XPS survey after O_2 annealing treatment

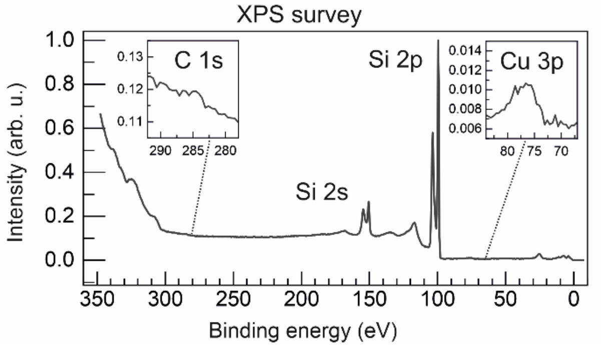

Figure S1. XPS survey after O_2 annealing treatment. This treatment removed the adventitious carbon and any residual polymer from the NP synthesis. The photon energy used was 400 eV which provides a surface-sensitive probing of the sample. C 1s and Cu 3p regions are shown with rescaled intensity in the inset.

LEEM blurring

The diameter values, measured in LEEM, of the NPs before and after the O_2 annealing treatment are slightly larger than the height measured by AFM, despite the spherical shape of the NPs. An explanation for this comes from the depth of focus of the microscope not covering the height of the NPs. Hence, it is impossible to keep the entire NP in focus. The consequence is the blurring of the NPs, resulting in larger NPs sizes measured in LEEM. Because this blurring is not only a function of the instrument (magnification, contrast aperture, kinetic energy of the probing electrons) but also of the actual size of the NPs, a secondary effect happens: an increase in the spread of the particle size distribution (Figure 1f in comparison to 1b). In other words, a 12 nm NP can have an apparent size of 20 nm (8 nm blurring), while a 20 nm can appear as 33 nm (13 nm blurring). Another relevant aspect to consider is that the morphology and the work function difference of the NPs can locally distort the electric field applied to the surface, causing an extra blurring in the image of the backscattered electrons. Thus, we have provided in the paper not only the LEEM-determined initial particle sizes, but those obtained by AFM (height, since the diameter also suffers significant artifacts associated to the size of the tip apex) and SEM.

In-situ reduction of copper nanoparticles

XPEEM composite image – Color and contrast stretching

Our XPEEM composite images in Figure 2 aim to represent the information in the NEXAFS spectra qualitatively and quantitatively. Qualitative by representing the elemental (Copper) distribution in the image and the oxide species (CuO and Cu_2O) in the sample. This is achieved by the colors green, magenta, and black. Quantitative by showing the amount of each species in each NP. The intensity of each color does this. Brighter means more of a given species (as observed in the NEXAFS spectra), while darker means less. The data acquired through our microscope correspond to a 16-bit range (minimum pixel value = 0, maximum pixel value = 65535). Yet, the actual range of pixel intensities is significantly less than the theoretical maximum. For visualization purposes, contrast stretching is essential. In monochrome images, contrast stretching makes the pixel with the highest intensity appear 100% white, while the lowest appear 100% black. Combining two images with different histograms can be challenging when the histograms have different distributions, as shown in Figure S2j. Three possibilities that keep the histogram linear are possible: i) using the minimum and maximum of the combined histogram, Figure S2a-c, ii) using the minimum and maximum of each channel, Figure S2d-f, and iii) setting the minimum and maximum to facilitate the visualization and interpretation of the data, Figure S2g-i. In the set of XPEEM images taken after the 593 K annealing step, the first option did not result in an understandable composite image because some particles have a big intensity at the green channel, making the magenta channel appear entirely dark. The second option also misrepresents the NEXAFS spectra, making it appear that most of the NPs were reduced entirely to Cu_2O, which is not the case when integrating the stack of images and analyzing the NEXAFS spectra of the NPs. Therefore, we opted for the third option, selecting a minimum and maximum for each channel, making the combined image retain the information of the individual channels. Thus, the images are scaled to properly represent the information in the spectra, enabling the contribution of the two copper species to be appropriately distinguishable while avoiding saturating/clipping the final image. For the purpose of clarity, we add here the values of the histogram and set minimum, and maximum intensity for the Figure 2 XPEEM composite images. We also show an example of the composite image for a larger field of view, in Figure S3.

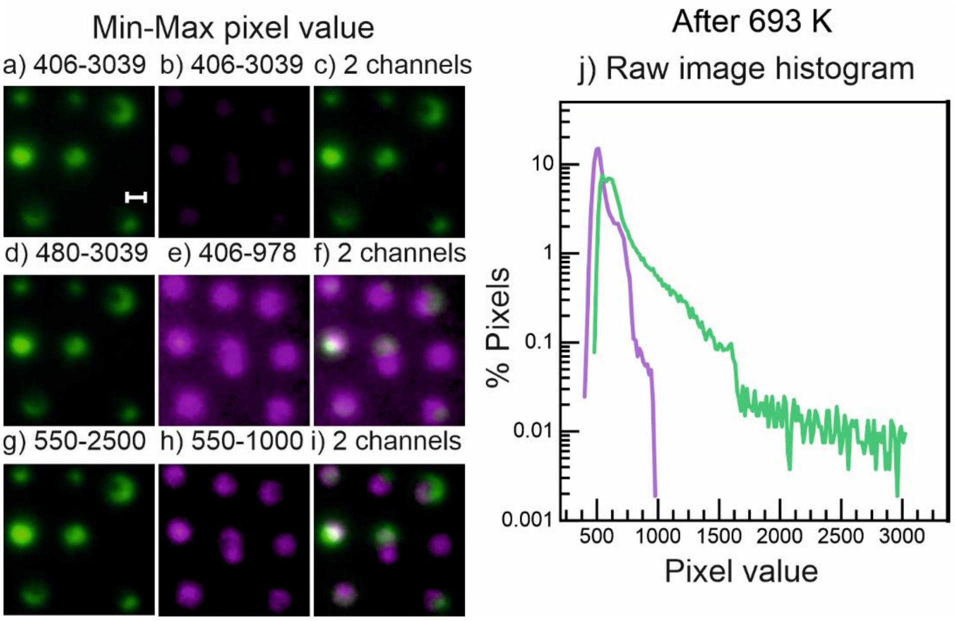

Figure S2. Histogram and contrast stretching of the same raw data. i) are data displayed in Figure 2.

	Green channel (CuO)				Magenta channel (Cu_2O)			
Temp(K)	Set Min	Set Max	Hist Min	Hist Max	Set Min	Hist Min	Set Max	Hist Max
307	714	714	2800	2961				
393	800	807	3400	3565				
453	750	744	3200	3394	700	597	1020	962
593	550	480	2500	3039	550	406	1000	978
643	260	208	1700	1802	260	189	750	729

Table S1. Contrast stretching details of Figure 2. Because no signs of Cu_2O were observed in the first two annealing steps, only the green channel was used to make the XPEEM image.

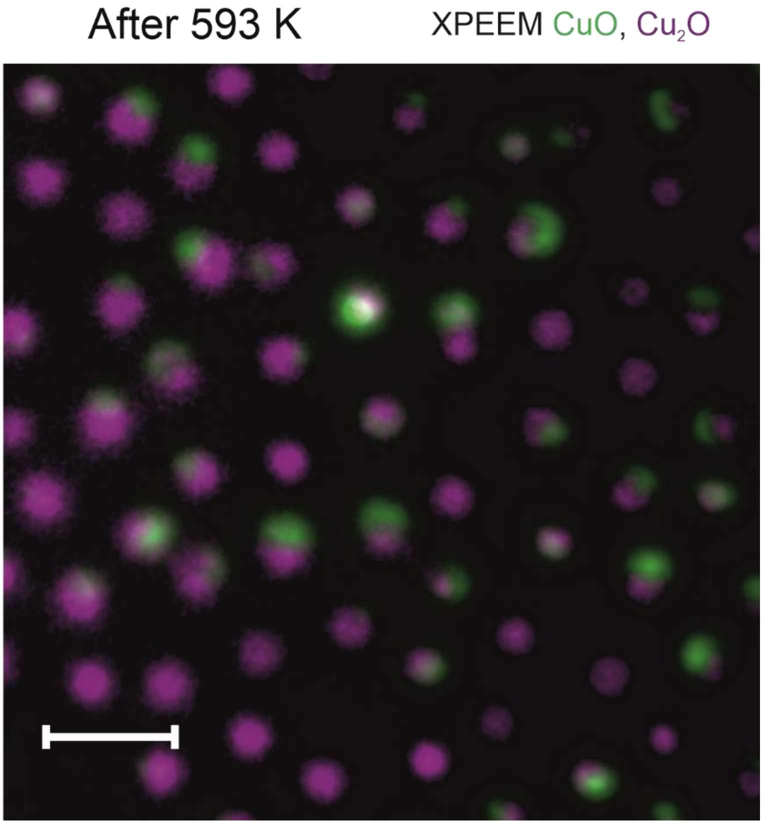

Figure S3. Extended view of the XPEEM image after the 593 K annealing treatment. The scale bar is 200 nm.

NEXAS peak intensity

One unusual aspect of the NEXAFS spectra in Figure 2 is the higher peak intensity for some particles. NP #4 has almost double the peak height as NP #7, consistently measured in the first three rows (Figure 2a-c). One explanation comes from a morphological feature, such as the geometry or size of the NPs, affecting the volume probed. The peak height intensity correlation with the LEEM NP size is demonstrated in Figure S4, which shows that the bigger the NP size is in LEEM, the higher the peak intensity is in NEXAFS. Another contribution could originate from different chemical compositions, such as a damping element encapsulating and affecting the peak intensity at different NPs. However, this contribution is harder to pinpoint, considering that there is Si XAS signal coming from the NPs region (Figure S5), and it is not possible to discern if the signal is coming exclusively from the top, sides, or less likely bottom of the NPs.

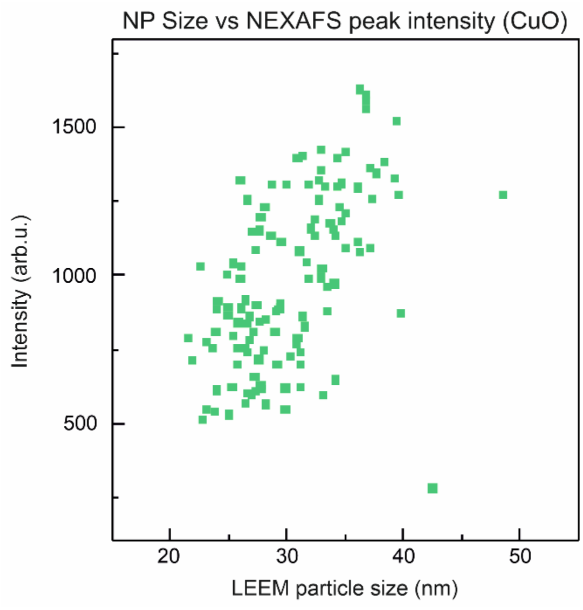

Figure S4. XPEEM-intensity dependence on NPs size. The NPs XPEEM intensity is taken before the annealing steps in UHV; the NEXAFS peak energy is at 931 eV. The intensity is corrected by subtracting the pre-edge baseline. The bigger the NP, the higher is their XPEEM intensity, with the Pearson correlation coefficient equal to 0.57

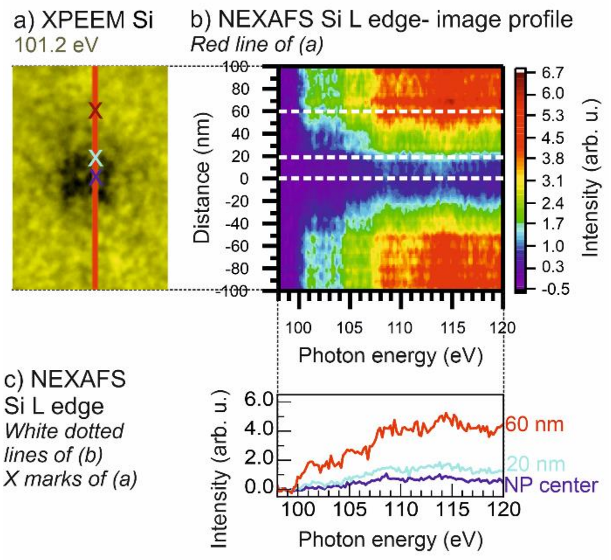

Figure S5. NEXAFS of the Si L edge of the NPs and the substrate nearby. By taking a line profile, red line in a), across one NP over the energy range of the Si L edge, one can observe the b) NEXAFS spectra at every point, starting from the center of one NP and 100 nm away from it. The dotted lines of b) correspond to the spectra shown in c), where the intensity of the substrate is higher than the NP.

Normalization of XPEEM images/spectra

One of the aims of XPEEM imaging is the chemical mapping of the sample. However, the contrast in a PEEM image is also influenced by other contributions and can become quite complex to analyze, depending on the sample. These other contributions include shadows caused by grazing illumination of morphologic features (objects with big height), the laterally inhomogeneous X-ray beam intensity, energy gradients over the observed field of view, and the XPS background of every peak excited from the photon energy used for the measurement. To remove these contributions, data treatment must be performed. Regarding the analysis of NEXAFS data generated from secondary electrons, one can use the pre-edge intensity to normalize the data. It is possible to divide or subtract the image at the peak of interest by the image at the pre-edge. Such subtraction will remove the background leading to the peak, while the division will remove beam inhomogeneities and shadows (Figure S6). In the main manuscript, we used either correction depending on the circumstance. For spectra, we divided, while for images, we used subtraction for Figure 6, and no correction for Figure 2, since the X-ray beam was homogenous in the displayed area.

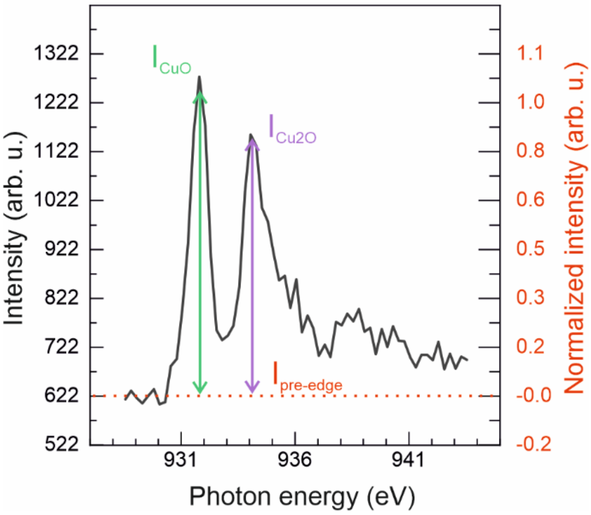

Figure S6. Normalization of the NEXAFS spectra. To use the intensity of the NEXAFS spectra as a parameter that quantifies the content of each species, we need to take into account the differences in the overall signal across different NPs. These arise from the beam intensity not being homogeneous across the field of view (FoV). In other words, the photon flux is different for the middle of the image than the sides, for instance. Normalizing by dividing the spectra by the pre-edge intensity (red-dotted-line) removes this effect. From all spectra a factor 1 was subtracted to leave them spectra starting at y = 0, for practicality purposes.

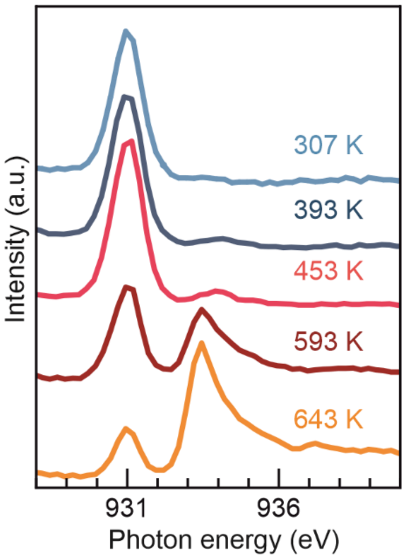

Figure S7. Average NEXAFS signal of every NP. Each spectrum shown is an average, derived from aggregating the individual NEXAFS spectrum of each of the NPs visible in the field of view. All spectra were generated from the same NP set, in other words, the same region, taken after each annealing step. Notice that this is different from an integral spectrum, where the entire image would contribute to the signal, here only the NPs are responsible for the signal.

NEXAFS data expressed as a *f* variable, and the importance of weighting factor

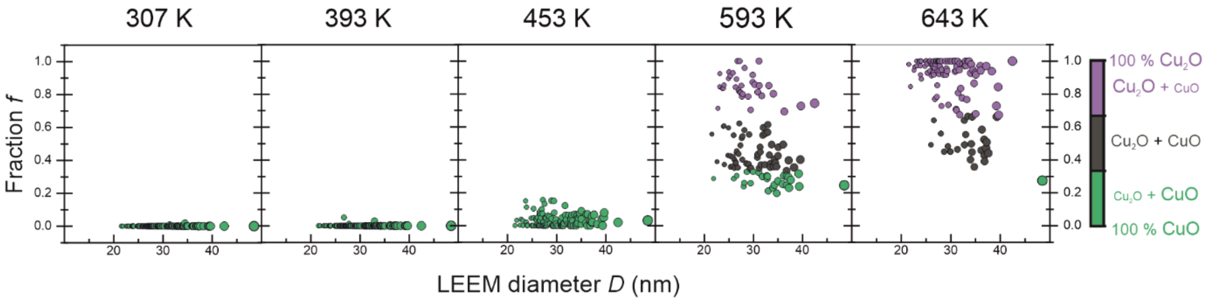

Figure S8. The progression of the converted fraction f against the NP diameter D measured in LEEM. The complete set of annealing temperatures of Figure 3c.

Before correlating structure (LEEM) to chemical data (NEXAFS/PEEM), one should apply a reduction to the NEXAFS data to derive meaningful physical and chemical insights. As shown in Figure 2, in the entire annealing experiment, only two components—CuO and Cu_2O— are visible in the NEXAFS spectra. Considering the nature of the experiment, annealing in UHV, and the spectral changes observed, we can say that we have a reduction from CuO to Cu_2O. This reduction reaction can be written as 2CuO → Cu_2O + O or $Cu^{2+} + e^{-}$ → Cu^{+}. Consequently, we can now express the reaction progression as a fraction *f*, defined as the fraction of Cu^{2+} converted to Cu^{+}. With other words:

$$f = \frac{N^{(+)}}{N^{(+)} + N^{(2+)}} \qquad (eq\ 1)$$

Whereas $N^{(2+)}$ and $N^{(+)}$ are the numbers of Cu^{2+}, and Cu^{+} atoms in a nanoparticle. The total number of Cu atoms (N) in a NP remains constant during conversion $N = N^{(+)} + N^{(2+)}$. Since the Cu L_3-edge NEXAFS spectra of both CuO and Cu_2O are characterized by single absorption peaks at 931 eV for Cu^{2+}, and 933.6 eV for Cu^{+}, it is possible to use the peak intensities (amplitudes) to quantify the amount of each species in a NP. This is possible, because they are proportional: $I^{(2+)} \sim N^{(2+)}$, and $I^{(+)} \sim N^{(+)}$. Also, for simplification purposes, the peak intensities described in the remaining of the paper are already normalized by a subtraction of the pre-edge intensity.

Subsequently, as the reduction reaction progresses, the intensity of the Cu^{2+} linearly decreases from initial $I_{max}^{(2+)}$ down to 0, whereas the Cu^{+} increases. To put it clearly:

$$I^{(2+)} = (1-p) I_{max}^{(2+)} \qquad (eq\ 2)$$

$$I^{(+)} = p\, I_{max}^{(+)} \qquad (eq\ 3)$$

Whereas $I_{max}^{(2+)}$ and $I_{max}^{(+)}$ are the peak intensities for the two oxide species for the non-reduced and the fully-reduced case, respectively, and the $I^{(+)}$ and $I^{(2+)}$ are the peak intensities during the reduction when a portion of *f* is already converted.

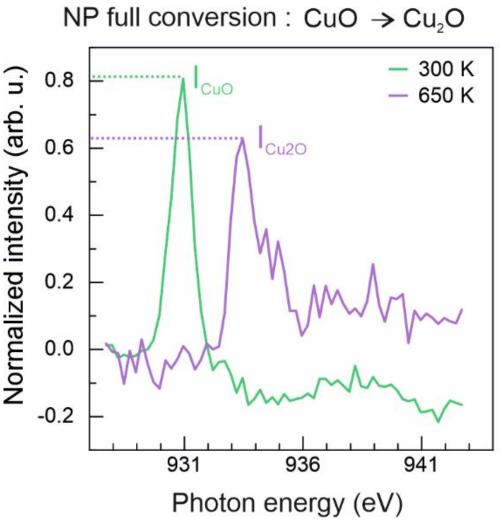

Figure S9. Estimation of γ factor. Spectra showing a NP which had a full conversion from CuO to Cu_2O after the annealing steps. The peak intensity of the NEXAFS spectra decreased after the conversion, signalizing the need for a weighting factor which ensures that the weighted sum of the two peak intensities is constant during the conversion and equal to the final intensity.

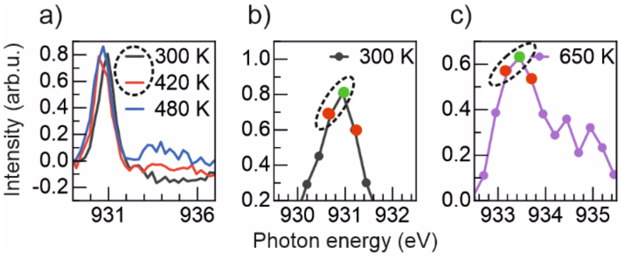

Figure S10. CuO signal, and dividing by the sum of the intensities at both CuO and Cu_2O edges. If this value v is more than 95% we considered that no conversion happened, and considered the peak intensity at this annealing step valid for the calculation of the maximum $I_{max}^{(+)}$. In a) the spectra obtained at RT, and after 420K, show no Cu_2O, therefore are counted for the calculation of $I_{max}^{(+)}$. The spectrum after 480K, had a value of 0.87, which can be observed in a larger than noise peak appearance at the Cu_2O region (933.8 eV). Therefore, this spectrum didn't count for $I_{max}^{(+)}$, since it also contains a Cu_2O contribution. Analogously, a full conversion is defined when v is lower than 5%. As can be seen, the normalized intensity of a given NP fluctuates little across the different annealing steps, which is a further indication that the NEXAFS intensity is correlated with the morphology of this NP. In fact, when taking in account only the NPs which did not convert in the first stages, 37 NPs, the average of the standard deviation of those is equal to 0.07. This low value shows that the intensity of the NEXAFS edge remains fairly constant across the annealing steps for a given NP. b), and c) show how the intensities of CuO ($I_{max}^{(+)}$), and Cu_2O($I_{max}^{(2+)}$) are obtained. First, the peak position is determined for every NP. Then, the mode is calculated, which is necessary, because not all the NPs have the same peak position, due to a gradient in photon energy in the same FoV (3.11 μm) of the order of 1 photon-step-size (x axis). This means that the spectrum is shifted in the x axis when you compare a NP in the very extreme right of the FoV to the one in the other extreme. After the mode is determined (marked as a green dot), we check for the neighbor dots (red dots), inside 1 photon-step-size range. We average the value of the mode, with the highest intensity neighbor dot. This serves to counter the photon energy gradient effect, and the general noise expected in measuring a single NP spectra. While the intensity of CuO($I_{max}^{(+)}$) is averaged between the intensity of up to the first three annealing steps, the c) Cu_2O($I_{max}^{(2+)}$) is obtained from the last annealing stop only (after 650 K), where most NPs achieved full reduction. The γ value for a single NP is finally calculated by dividing the $I_{max}^{(+)}$ by the $I_{max}^{(2+)}$. We than average these values for the 50 NPs which had a full conversion, and the final γ was 0.84.

During the conversion, the initial intensity $I_{max}^{(2+)}$ of the Cu^{2+} peak is completely transformed into the intensity $I_{max}^{(+)}$ of the Cu^{+} peak, whereas the relation of the two intensities is given by $I_{max}^{(+)} = \gamma\, I_{max}^{(2+)}$, with γ as a weighting factor, experimentally determined as $\gamma = 0.84$ (Figure S9, Figure S10). This factor eliminates some issues, such as a possible difference in cross section between the two species, and the fact that we are using the intensity of a peak, and not the area under the spectra as a descriptor.

Since the NEXAFS edges of both species do not necessarily have the same peak shape, or full width at half maximum, by applying this factor γ, we can circumvent this issue. Therefore, the weighted sum of the two peak intensities is constant during the conversion and equals the final intensity.

$$I^{(+)} + \gamma\, I^{(2+)} = I^{(+)}_{max} = const. \quad \text{(eq 4)}$$

Eq. 4 inserted in **eq. 3** yields an equation for the converted portion based on experimental intensities:

$$f = \frac{I^{(+)}}{I^{(+)} + \gamma\, I^{(2+)}} \quad (eq\ 5)$$

NP morphology and oxidation state

Relevance of the position of NPs

With the coordinates of the NPs, we have access to some degrees of information. On the first level, the position of the NP can have an indirect effect on the reduction pattern observed. For instance, the presence of an impurity, defect, or a chemical difference in specific spots of the substrate could enhance or inhibit the reduction reaction. On a second level, the presence of more, or less NPs in a given region can affect the kinetics of the reaction, especially if the reaction depends on diffusion through the substrate. On a third level, the distance between two neighbored NPs can influence a given chemical reaction, generally speaking. This is the case of tandem catalysts, where two different sites present on two different NPs, can be responsible for two different consecutive chemical reactions, where one NP produces B from A, and the second NP produces C from B. The distance between the NPs could then have an influence on the production of C, if the intermediate product B migrates through the substrate.

Data binning, front direction, and NEXAFS simulation

The lower correlation (size vs. *f*) observed in the main manuscript before binning could have a physical meaning. This is because the combination of the reaction mechanism and the technique used to probe the system can provide experimental *f* values distinct from reality on a NP level.

First, we will discuss the possible mechanisms of supported NP reduction. Two prominent families of reactions exist.[1] Solid-state diffusion reactions involve the diffusion of a chemical species through the bulk of one of the solids involved. One example is the interaction of transition metal oxides with alumina, in which spinel structures or solid solutions of the transition metal in the alumina are formed.[1] However, we did not observe, in the temperature range studied, a significant drop in the copper XPS signal. This signal decrease would be one of the ways to detect Cu diffusion into the bulk of the Si substrate. Neither a secondary species was formed, such as copper silicides.[2] The most significant change was the transition from CuO to Cu_2O. Therefore, this mechanism seems unlikely to be the case here. On the other hand, the second family of reactions are interface-controlled (IC), which means that interfacial processes limit the reaction kinetics. In these reactions, atoms at the surface have a higher probability of reacting than bulk atoms. Interface-controlled reactions can be of two types. In the first type, all the surface of the NP reacts at the same time, creating a continuous interface that expands inwards. In this case, the PEEM pseudo-color images shown in Figure 2 would be single-colored. There would be no contrast, because the entire surface would be reducing at the same rate. In the second type, the nucleation of the reduced phase occurs at specific points of the surface. For instance, the nucleation would start at a defect or the interface between the substrate and the NP and then progress unidirectionally across the NP.[3] Figure S11 shows a strong case for the latter, where a gradient in the oxidation state is observed across a single NP. Coupling this information with the time evolution of the reduction fronts displayed in Figure 2, with a unidirectional progression, the most likely mechanism of this NP's reduction is the nucleation-controlled interface reaction. It should be noted that we did not observe multiple nucleation points in the same NP.

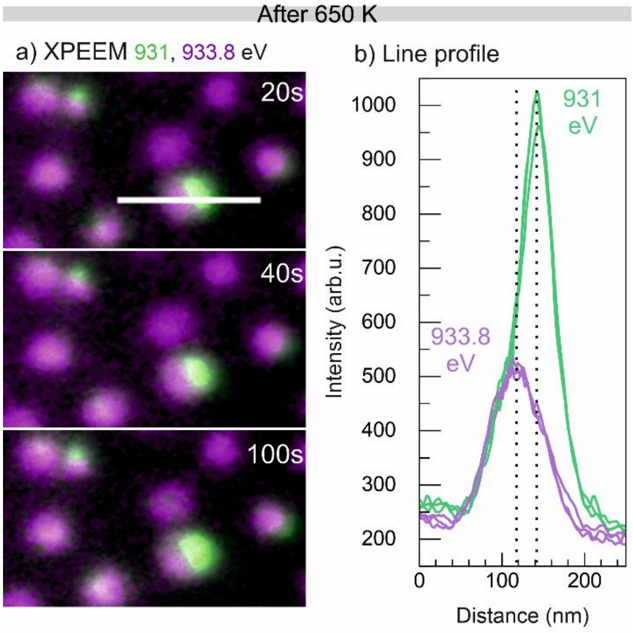

Figure S11. Reaction front on single Cu NPs. Composite XPEEM images were taken after cooling down from the 650 K annealing step, at two different photon energies, near the CuO L_3 edge (green) and Cu_2O L_3 edge (magenta). Each image in the composite has a 20 s acquisition time. For this data, no correction by the pre-edge was performed. A consistent gradient in the oxidation state is observed, strongly indicating the presence of a reaction front. Different NPs have gradients in different directions. A white profile line with a length of 250 nm traced in the same position for each image shows b) three almost similar curves for each energy, highlighting how this gradient is consistent and not a result of experimental artifacts.

Now that we have established that the reaction is propagating in a front manner, in a single direction, we should determine in what direction this front is moving, and how to measure this front. The height and therefore diameter of the spherical NPs are in the range between 8.1 and 20.5 nm. The typical IMFP length λ of the produced secondary electrons is between 2 and 3 nm, which means the information (sampling) depth $L = 3\lambda$ is about 6 to 9 nm, from which 95% of the detected signal originates (Figure S14).[4] Because the particles are higher than L, the signal from the bottom of the NPs cannot be detected. This means that under specific front directions (Figure S13), the signal we are detecting, and interpreting as f, is far from the real volume of the NP converted to Cu_2O. However, if one assumes that all possible configurations of the reduced domains are possible, by rotation, then the averaging over the particles of the same size may yield the correct portion (Figure S12). To test this hypothesis, we coded a program (Figure S15) that simulates the f value that we would get experimentally, and compared to the theoretical f value, which is the ratio of the converted volume of the NP (to Cu_2O) over the total volume. To overcome a problem that would be quite hard to solve analytically, we divided a NP (2D or 3D) in a given number of infinitesimals (defined by the user), arranged in either random (Monte-Carlo), or quasi-random fashion (Sobol) (Figure S16). Each point (infinitesimal) contributes to the detected signal with a given intensity that depends on the depth (d) that this infinitesimal is located, and the inelastic mean free path, which is defined in our case as a ratio of the NP radius. The signal intensity for each point is:

$$I = \mathrm{e}^{-\mathrm{d}/\lambda} \quad \text{(eq 6)}$$

By integrating the intensity of each point for each phase, and dividing by the total intensity, one can get the converted fraction f. This program, therefore, can be used by anyone who faces a similar situation, a reaction moving unidirectionally across a NP. The key takeaway is that averaging two opposite (vectors) moving fronts can indeed decrease the error margin of the measurements (Figure S13). For instance, our NP with an average height of 14.5 nm would appear reduced with a f value of 0.64 (64% conversion), even if the geometric/theoretical conversion (f_{Th}) was only 25%, if the front was moving from the top to the bottom of the NP. By measuring enough NPs of the same height, one can negate these isolated readings, and achieve a result, in which f is almost f_{Th}, as displayed in the mean curves (green) in the graphs of (Figure S13). Therefore, binning the NPs is a good strategy to counterbalance the multidirectional front direction, and inelastic mean free path limitations.

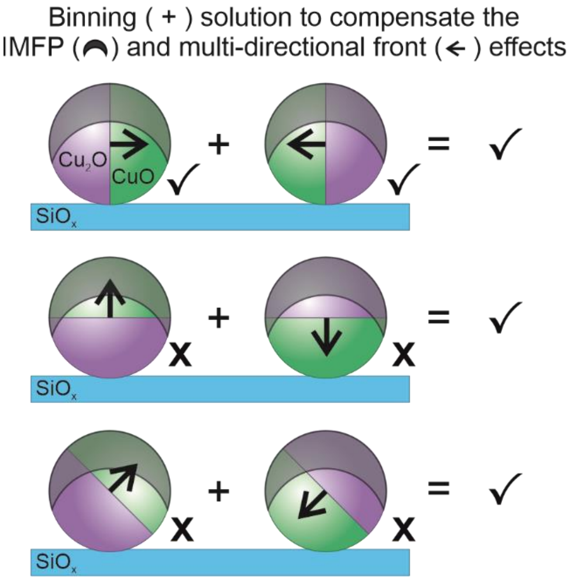

Figure S12. Binning hypothesis. This schematic shows how the IMFP *affects the measurements. The most evident case is when the reaction front moves perpendicularly (in relation to the substrate). For a conversion of half of the volume of the NP, there can be two completely different readings. One is that the NP is entirely CuO, and the other is entirely Cu_2O. By averaging the signal of different NPs of the same height, we hope it is possible to remove this effect.*

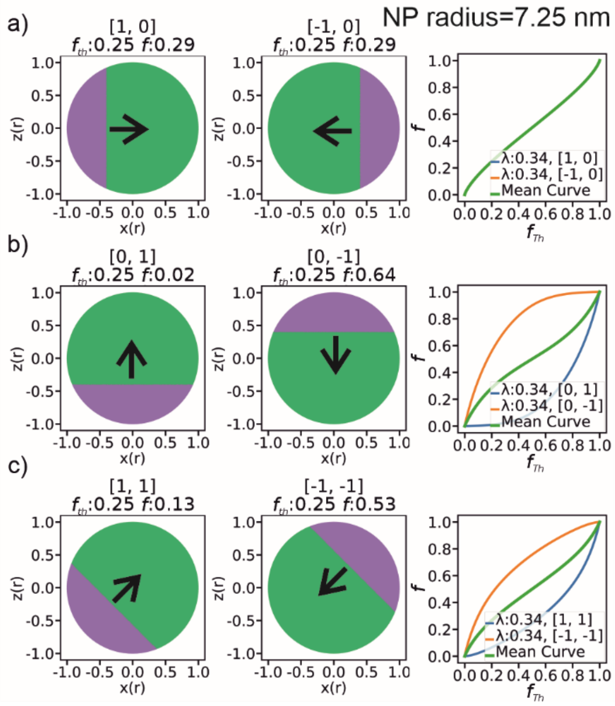

Figure S13. Front direction influence on the f value. The direction that the front propagates influence the measured f converted volume. The theoretical f value (f_{Th}), which is the percentage of the geometric volume converted from the first phase (green, CuO) to the second phase (magenta, Cu_2O) is fixed in these images, 25%. Depending if the front propagates parallel to the substrate, or perpendicularly, the measured f values are distinct. However, averaging two opposite moving fronts, can mitigate the deviation of *f from f_{Th}. This highlights the importance of binning, or averaging, a significant amount of NPs of the same height.*

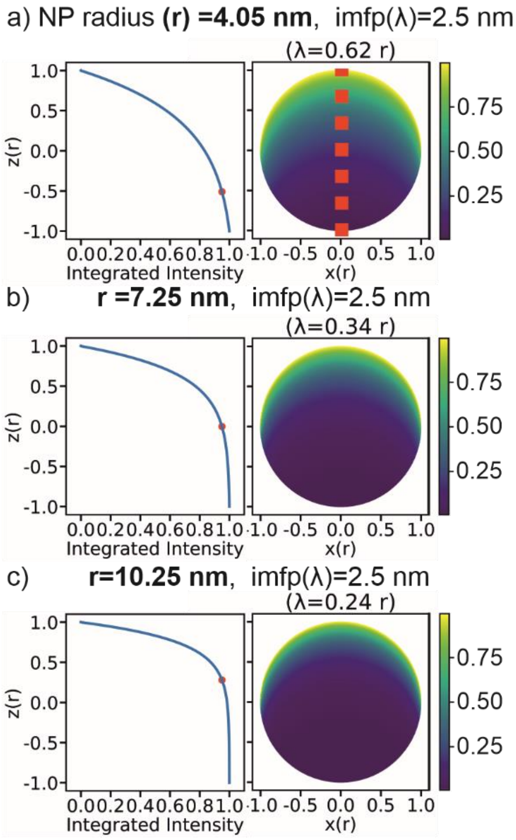

Figure S14. Information depth for different NPs. a) small NP in AFM, b) average, and c) big. We simulated the signal coming from the center of a NP, represented by the red line, more precisely between -0.1 and 0.1 x (r). The graphs on the left show the z axis of the NP in terms of radius versus the cumulative intensity. The red point represents the information depth, the depth where you have 95% of the total signal of your sample. The figure shows that for the same inelastic mean free path, but different NPs heights, you have different surface sensitivity across the NPs. While in smaller NPs a), the signal comes almost from the entirely NP, so bulk sensitive, in the bigger NPs, c) most of the signal originates from the surface.

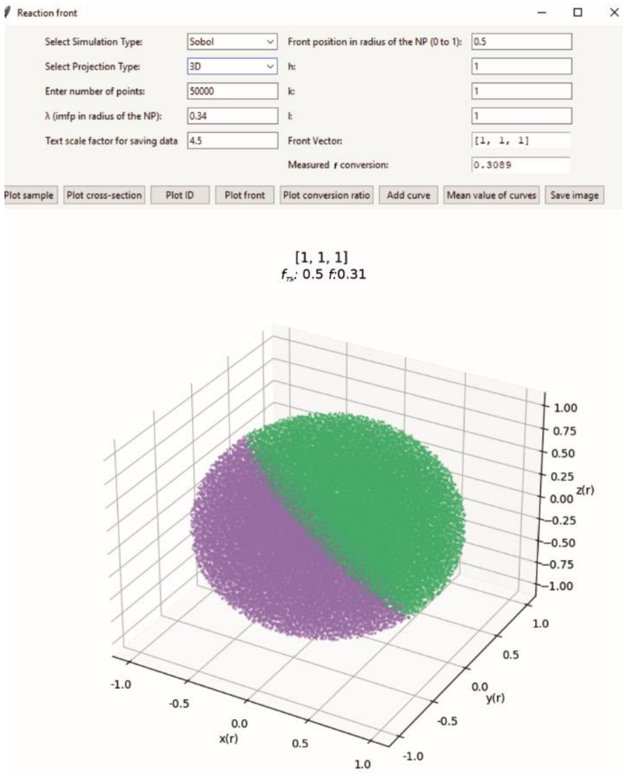

Figure S15. Interface of the simulation program. Parameters such as IMFP, and front direction can be chosen to simulate the f (simulated detected converted fraction), and f_{Th} (Theoretical/geometric converted fraction) values.

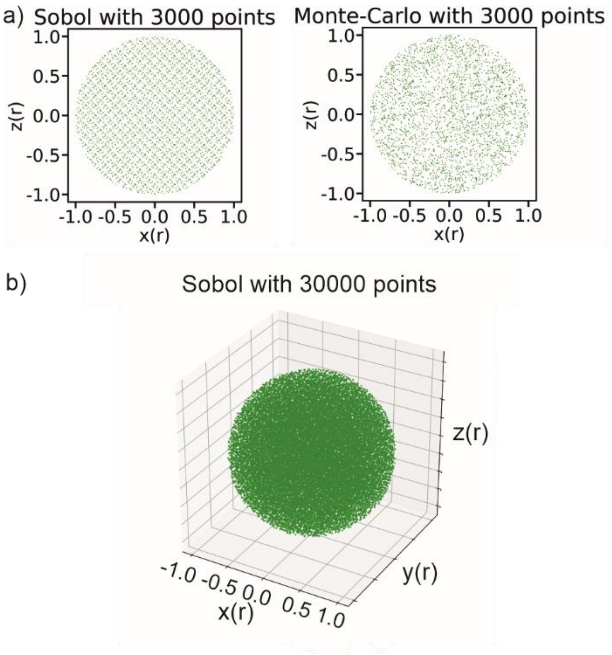

Figure S16. Simulation options. The NP can be divided in any number of points, defined by the user, in this case 3000 for a), and 30000 for b). The higher the number, the more statistically sound is the final calculation. The schematic in a) presents the two different options to distribute the points (infinitesimals), either with a quasi-random (Sobol) or random simulation (Monte-Carlo). The plot in b) shows that the program can also calculate in 3D space. For easy of visualization, the calculations in the rest of this paper are in 2D, using a Sobol simulation with 500000 points. In the 2D case, the z axis is perpendicular to the substrate, in other words, normal to the surface, or parallel to the detector axis. The data in b) are displayed with an equal ratio, therefore y, and z axis also are showing -1.0 to 1.0 length in radius.

Start D	End D	Bin size	Mean D	n NPs
21.6	23.5	1.9	22.5	5
23.5	25.4	1.9	24.6	13
25.4	27.3	1.9	26.5	21
27.3	29.3	1.9	28.1	17
29.3	31.2	1.9	30.3	14
31.2	33.1	1.9	32.2	16
33.1	35.0	1.9	34.1	16
35.0	37.0	1.9	36.3	8
37.0	38.9	1.9	37.5	5
38.9	40.8	1.9	39.4	4
40.8	48.5	7.7	45.5	2

Table S2. Description of the bins used to group the NPs by size. The mean diameter D is the mean value of every NP diameter inside a given bin, and not the proper center of the bin. We opted to group the last two bigger NPs in a single bin to increase the reliability of the reading, given that a single NP is prone to display big errors. Every size is given in nm.

Equation for the temperature-dependent front velocity

With a NP containing a single reaction front, it will have two regions during conversion: a region converted to Cu_2O with a volume $V^{(+)} = N^{(+)}\, n^{(+)}$ and a non-converted CuO region with a volume $V^{(2+)} = N^{(2+)}\, n^{(2+)}$. With n being the Cu concentration of each species. Inserting these equations in (eq 1) yields:

$$f = \frac{V^{(+)}/n^{(+)}}{V^{(+)}/n^{(+)} + V^{(2+)}/n^{(2+)}} = \frac{V^{(+)}}{V^{(+)} + \frac{n^{(+)}}{n^{(2+)}} V^{(2+)}} \qquad \text{(eq 7)}$$

The Cu concentrations of CuO and Cu_2O are $n^{(2+)} = 52.7$ nm^{-3} and $n^{(+)} = 50.7$ nm^{-3} and therefore, differ by only 4 % which we can neglect in the following. We assume that the reduction reaction occurs as a propagating front with a constant but temperature-dependent velocity v. If $\Delta V^{(+)}$ is the volume reduced from Cu^{2+} to Cu^{+} by the temperature treatment at T for the time Δt, then this volume can be calculated by:

$$\Delta V^{(+)} = A\, \Delta s = A\, v\, \Delta t \qquad \text{(eq 8)}$$

Here, A and Δs are the areas of the reaction front propagating by the length Δs. The temperature dependent front velocity v is defined as $v = \Delta s/\Delta t$. Summing up all temperature treatments (labelled with index i, whereas T_i is the temperature of the i-th treatment yields in the following sum:

$$V^{(+)} = \sum_i A(T_i)\, v(T_i)\, \Delta t_i \qquad \text{(eq 9)}$$

Inserting this in (eq 7) results in:

$$f = \frac{V^{(+)}}{V} = \frac{\sum_i A(T_i)\, v(T_i)\, \Delta t_i}{V} \qquad \text{(eq 10)}$$

Taking the difference of the f values of subsequent temperature treatments at T_i and T_{i-1}:

$$\Delta f_i = f(T_i) - f(T_{i-1}) = \frac{A(T_i)\, v(T_i)\, \Delta t_i}{V} \qquad \text{(eq 11)}$$

From this, one can easily derive the temperature dependent velocity:

$$v(T_i) = \frac{V}{A(T_i)} \frac{\Delta f_i}{\Delta t_i} \qquad \text{(eq 12)}$$

Front area changes due to geometry, diameter, and *f*

Depending on the shape (*Figure S17*) of the reduced material, the area A can change during the propagation of the front, except for simple cases like cubes or rods where the ratio V/A is constant, for instance a cube has $V/A = D$ with D being the length of the cube, and for a rod, $V/A = l$. With l being the length of the rod, in the scenario of the front propagating along the rod axis. For complex shapes, such as a sphere of diameter D, the calculation of the front velocity must take in account the variable area of the front (*Figure S17*). For instance, at half conversion, the front area will be at the maximum, while at the beginning it will be at its minimum. Therefore, A depends not only on the diameter D of the sphere but also on the value f. Two approaches can be taken to arrive to A, one is simulating the area of the front for a given f in our program, and another is within a good approximation of better than 2% (Figure S18), through this formula:

$$\frac{V}{A(T_i)} = \frac{2}{3} D \frac{1}{g(f)\left(1 + \frac{1}{8}(1 - g(f))\right)} \qquad \text{(eq 13)}$$

With $g(f) = 2\sqrt{f\,(1 - f)}$.

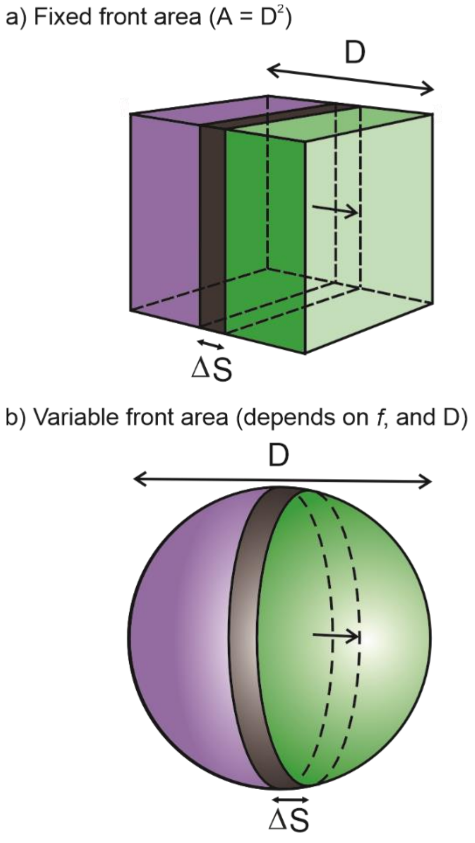

Figure S17. Geometry influence on the front velocity calculations. While the front velocity can be easily calculated in the case of nanocubes, given the constant front area in a), for nanospheres the calculation is more complex, because the area of the front depends not only on the NP size, but also in the conversion f. This area can be calculated either through simulation (such as using our program), or through an approximation.

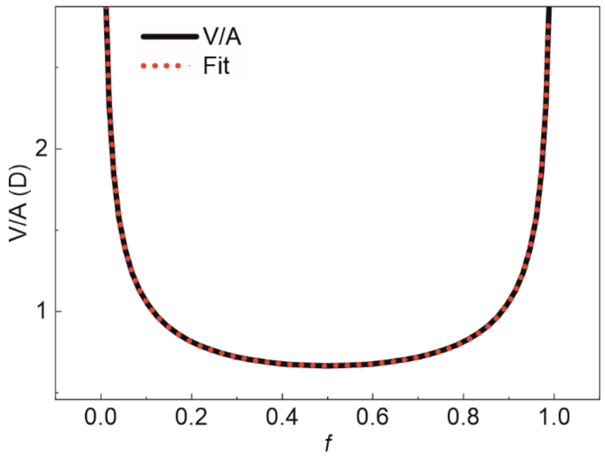

Figure S18. Ratio V/A dependence on f. In red the analytic fitting function, that is within 2% the ratio V/A.

Correction of missing measurement of the annealing step 523 K

For the 523 K annealing step, due to technical issues there is no PEEM image, and consequently no Cu L_3-edge NEXAFS at the same region (same NPs). Thus, we don't have the *f* values for this annealing step. This affects the Δf of the following annealing temperature (593 K), in Eq 12. Therefore, for the calculations of Figure 4, we had to apply a correction to offset this issue. We will interpret that the reaction ran longer for the annealing step immediately after the 523 K, so 593 K. Instead of the real 10 minutes at the annealing temperature, we will use the value 13 minutes. The idea is that 3 minutes of the reaction at 593 K would be comparable to 10 minutes at 523 K.

Arrhenius plot, and apparent activation energy

The reduction reaction of CuO to Cu_2O can be written as:

$$2CuO \overset{k_+, k_-}{\Longleftrightarrow} Cu_2O + O$$

with the reaction coefficient k_+ and k_- for the back reaction. The "free" oxygen diffuses towards the surface of the NP where it desorbs with a rate described by the coefficient k_{des}. In many diffusion limited reactions, propagation reactions fronts have been observed.

As predicted by Luther in 1906,[5] the velocity v of a propagating reaction front can be described by

$$v = C\sqrt{Dk}$$

with a dimensionless constant C and the diffusion coefficient D (in our case diffusion of oxygen through the copper oxide) and the rate-limiting coefficient k (in our case either k_+ or k_{des}).

Both, D and k show an exponential dependence on the temperature with specific activations energies:

$$D = D_0\, e^{-\frac{E_{diff}}{k_B T}}$$

$$k = k_0\, e^{-\frac{E_{react}}{k_B T}}$$

with the diffusion energy E_{diff} and the reaction barrier E_{react}.

Therefore, also the velocity shows an exponential dependence:

$$v = C\sqrt{Dk} = v_0\, e^{-\frac{E_{app}}{k_B T}}$$

with a so-called apparent activation energy $E_{app} = \frac{E_{diff} + E_{react}}{2}$ and $v_0 = CD_0k_0$.

If we now display the temperature dependence of our experimental velocity data in an Arrhenius plot (*i.e.* $\ln(v)$ versus $1/T$), then the slope of the graph will gain the apparent activation energy E_{app}.

It is worth mentioning that in most cases, the diffusion energy (~100 meV) is smaller than the reaction energy (~ 500 – 1000 meV). Therefore, we get the known approximation that the apparent activation energy is about half the barrier height of the rate-limiting step:

$$E_{app} \cong \frac{E_{react}}{2}$$

Substrate chemical state influence on the NP oxidation state.

Alternative reduction mechanism

A less likely mechanism could explain the CuO reduction. In this alternative scenario, the reduction of the NPs is hindered in the initial state due to the presence of an ultra-thin encapsulating SiO_2 layer and, hence, increases the activation energy of the reduction process by blocking the O_2 desorption from the copper surface. In cases of strong metal-support interactions (SMSI), NP encapsulation by the substrate can happen, but it is typical for easily reducible supports, which is not the case here. Nonetheless, it has been reported by Yang *et al.*[6] on SiO_2-supported Co and Pt NPs due to the formation of Si at the metal-SiO_2 interface, when annealing at 750 °C, under different reduction environments. In our scenario, the X-rays might affect a possible encapsulating silica layer, reducing its size and oxidation state and thus the permeability for oxygen; consequently, the particles in the exposed areas can finally reduce *via* the reaction 2 CuO (solid)→ Cu_2O(solid) + 1/2 O_2(gas). This scenario us not very likely, because it would demand the presence of SiO_2 on top of the surface of the NPs before the annealing steps in UHV. This is unlikely, considering that the only treatments done at this stage were annealing in an oxidative environment (O_2), and O_2 plasma.

Compensation of beam damage by oxygen dosage

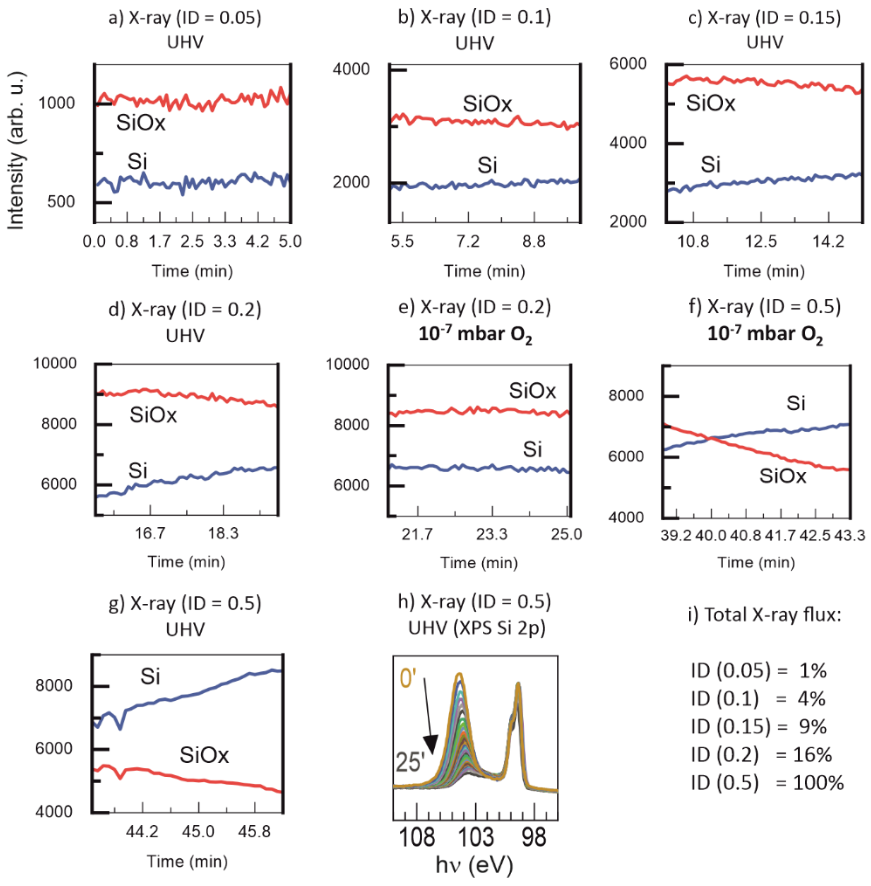

Figure S19. Effect of X-ray exposure in different conditions. In the initial minutes in UHV pressure, a) the insertion device (ID) aperture, measured in mm, is at the lowest size with an estimated X-ray flux of 1%, and the Si chemical species (Si and SiO_x) are stable, when opening the ID, thus increasing the flux, the SiO_x starts to decay, and the signal of Si increases with time, with a clear dependence with the total X-ray flux (a,b,c,d,g). When e) dosing O_2 in the 10^{-7} mbar pressure with an ID = 0.2 mm, the silica reduction stopped. However, by further f) opening the ID, the reduction continued, while the O_2 pressure (10^{-7} mbar) was maintained. The reduction process persisted when returning to g) UHV pressure. h) Shows the XPS at $h\nu$ = 630 eV of the Si 2p line, normalized by the Si peak, and i) shows an estimative for the total X-ray flux for each ID value.

NP morphology and oxidation state

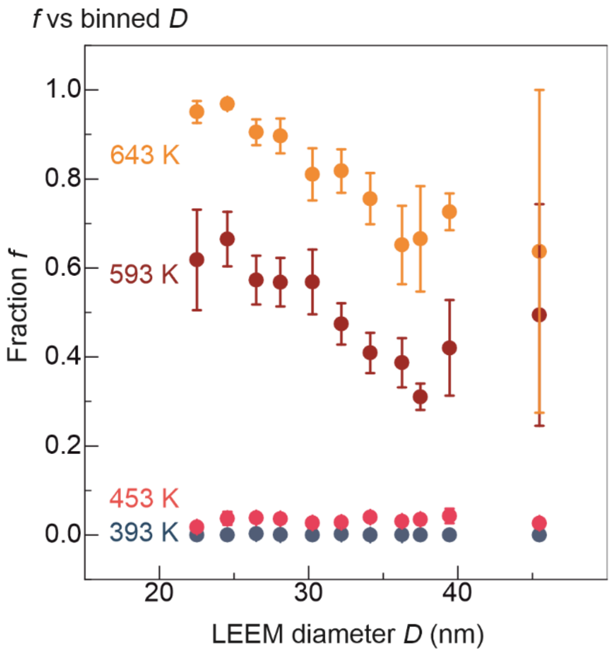

Figure S20. Average f ratios for NPs binned by their size. The complete data, including the standard error, of Figure 4a.

Experimental Section

Comparison with other spectro-microscopes

Technique	TEM & EELS	SEM & EDX	STXM	LEEM & XPEEM
Probe and detection	Electron in – electron out	Electron in – photon out	Photon in – photon out	LEEM: electron in – electron out<br>XPEEM photon in – electron out
Lateral resolution	High:<br><0.1 nm	High:<br><1 nm	Medium:<br>10 – 50 nm	Medium:<br>2 – 18 nm
Energy resolution	Medium:<br>~1 eV (EELS)	Low:<br>~ 100 eV (EDX)	High:<br>0.03 – 0.1 eV (NEXAFS)	High:<br>0.03 - 0.1 eV (NEXAFS)<br>0.2 eV (XPS)
Chemical information	Element and chemical state	Only element analysis	Element and chemical state	Element and chemical state
Surface sensitivity	Low:<br>100 nm - µm,<br>1 nm for cross-section (hard when *in-situ*)	Low:<br>100 nm - µm	Low:<br>100 nm - µm	High:<br>0.3 – 2 nm,<br>tunable (depth-profiling possible by varying hν/IMFP)
Beam damage	High	High	Medium	Medium
Energy range	High energy:<br>electrons: 100 – 300 keV<br>(no valence band mapping)	High energy:<br>electrons: 10 - 100 keV<br>(no valence band mapping)	Medium energy:<br>photons: hν = 100 – 1500 eV	Low to medium energy:<br>electrons: E_{kin} = 0 – 1000 eV<br>photons: hν = 100 – 1500 eV<br>Valence band spectroscopy (electronic state)<br>Core level up to E_b=~1000 eV (element, chemical state)
Sample requirement	Thin (< 100 nm)	No special requirement	Medium thin (100 nm – 5 µm)	Flat surface<br>(object height:width < 1:5)
Sample environment	Pressure range up to 1 bar (gas) or aqueous environment (cells with windows)	Pressure range up to 1 bar (gas) or aqueous environment (cells with windows)	Pressure range up to 1 bar (gas) or aqueous environment (cell with windows)	Pressure range from 10^{-10} mbar up to 10^{-5} mbar

Table S3. Comparison of LEEM/XPEEM with other spectro-microscopes. The strengths of LEEM/XPEEM are the high surface sensitivity, lower beam damage than the other microscopes and the accessibility of XPS core levels of catalytically relevant elements such as Cu, Pd, Fe, Ni, O, C and N, and their correspondent L, or K-edges for NEXAFS. Also, the standard base pressure of 10^{-10} mbar allows for the study of outermost clean sample surfaces. The strength of TEM, SEM and STXM is the possibility to study samples in gaseous and liquid environment, because special reaction cells with windows for the probing and imaged beam can be used due to the high mean free path length of the used electrons and/or light. Furthermore, TEM and SEM excel in outermost spatial resolution even down to the atomic scale.

REFERENCES

(1) Delmon, B. Formation of Final Catalyst. In *Preparation of Solid Catalysts*, 1999; pp 541-579.

(2) Benouattas, N.; Mosser, A.; Raiser, D.; Faerber, J.; Bouabellou, A. Behaviour of Copper Atoms in Annealed Cu/SiOx/Si systems. *Applied Surface Science* **2000**, *153*, 79-84. DOI: 10.1016/S0169-4332(99)00366-9.

(3) LaGrow, A. P.; Ward, M. R.; Lloyd, D. C.; Gai, P. L.; Boyes, E. D. Visualizing the Cu/Cu2(O) Interface Transition in Nanoparticles with Environmental Scanning Transmission Electron Microscopy. *J Am Chem Soc* **2017**, *139*, 179-185. DOI: 10.1021/jacs.6b08842.

(4) Powell, C. J. Practical Guide for Inelastic Mean Free Paths, Effective Attenuation Lengths, Mean Escape Depths, and Information Depths in X-Ray Photoelectron Spectroscopy. *Journal of Vacuum Science & Technology A* **2020**, *38(2)*, 023209. DOI: 10.1116/1.5141079.

(5) Luther, R. Propagation of Chemical Reactions in Space. *Elektrochem* **1906**, *12*, 596.

(6) Yang, F.; Zhao, H.; Wang, W.; Wang, L.; Zhang, L.; Liu, T.; Sheng, J.; Zhu, S.; He, D.; Lin, L.; *et al.* Atomic Origins of the Strong Metal-Support Interaction in Silica Supported Catalysts. *Chem Sci* **2021**, *12*, 12651-12660. DOI: 10.1039/d1sc03480d.
